# Identifying metabolic parameters as key indicators of hyperuricemia and ischemic stroke comorbidity via interpretable Clinlabomics models

**DOI:** 10.3389/fendo.2025.1737419

**Published:** 2026-01-13

**Authors:** Yao Jiang, Qin Li, Da Hu, Huaqiang Liao, Shu Chen, Hao Xu, Qian Wu, Mingcai Zhao, Jimin He

**Affiliations:** 1 Department of Clinical Laboratory Medicine, Suining Central Hospital, Suining, Sichuan, China; 2 Department of Information, Suining Central Hospital, Suining, Sichuan, China; 3 Faculty of Medical Technology, Shaanxi University of Chinese Medicine, Xi’an, Shaanxi, China; 4 Department of Neurosurgery, Suining Central Hospital, Suining, Sichuan, China

**Keywords:** Clinlabomics models, comorbidity, hyperuricemia, ischemic stroke, metabolic parameters, Shapley additive explanations

## Abstract

**Objective:**

Ischemic stroke (IS) with hyperuricemia (HUA) correlates with poor outcomes, yet the shared pathophysiological traits remain unclear. This study examined metabolic parameters in HUA-IS comorbidity and developed an optimal interpretable Clinlabomics model for risk assessment.

**Methods:**

A total of 2,164 IS patients and 2,459 healthy controls (HCs) were retrospectively enrolled. Participants were divided into four groups: HUA-IS (comorbidity, n=1,082), non-HUA IS (n=1,082), HUA HCs (n=1,314), non-HUA HCs (n=1,145); the latter three were defined as the non-comorbidity group. After 1:1 propensity score matching (PSM), 1,031 cases were matched in each group. Ten metabolic parameters were analyzed: serum uric acid at admission (SUA_admission), SUA on the third day of hospitalization (SUA_3d), triglyceride-glucose index (TyG), triglyceride (TG), high-density lipoprotein cholesterol (HDL−C), atherogenic index of plasma (AIP), atherogenic coefficient (AC), lipoprotein combine index (LCI), Castelli’s risk index I (CRI-I), and Castelli’s risk index II (CRI-II). Univariate/multivariate logistic regression, quartile-based logistic regression, and restricted cubic spline (RCS) analysis were used to explore parameters - comorbidity associations. Post-PSM data were split 7:3 into training/testing sets, least absolute shrinkage and selection operator (LASSO) regression selected features, and 11 machine learning algorithms developed Clinlabomics models. Additionally, the optimal model was validated in the testing set and an independent validation set.

**Results:**

After PSM, multivariate logistic regression identified AIP as the strongest risk factor (OR = 2.74, 95%CI: 1.80-4.19). The Q4 of TyG, TG, AIP, and LCI elevated comorbidity risk (*P* < 0.05). Besides, RCS showed nonlinear association of LCI with comorbidity (*P* < 0.05). The Recursive Partitioning and Regression Trees (rpart)-based Clinlabomics model exhibited favorable performance with F1-score, accuracy (ACC), and area under the curve (AUC) of 0.960, 0.960, and 0.986. At optimal hyperparameter (cp=0.0017), the model achieved AUCs of 0.987 (95%CI: 0.982-0.993), 0.955 (95%CI: 0.939-0.972), and 0.957 (95%CI: 0.915-0.999) in the training, testing, and validation datasets, respectively, correctly identifying 87.7% non-comorbidity and 98.0% comorbidity patients in validation. SHapley Additive exPlanations (SHAP) analysis identified UA_admission, UA_3d, TyG, TG, AIP and LCI as key metabolic indicators.

**Conclusion:**

TyG, TG, AIP, and LCI were critical metabolic parameters for HUA-IS comorbidity, which warrant heightened attention in future comorbidity research.

## Graphical Abstract

**Figure:**
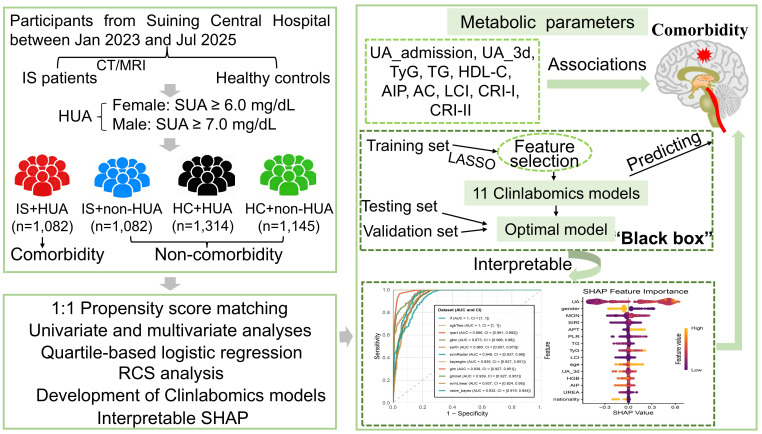

## Introduction

Stroke, a major acute cerebrovascular disease, ranks among the leading causes of death and long-term disability worldwide, imposing a substantial burden on global health. Based on estimates from the Global Burden of Disease Study 2021, it is projected that by 2025, disability-adjusted life years (DALYs) due to stroke will increase by 12.9%, and stroke-related mortality will rise by 28.5% (1), underscoring a pressing public health challenge. Ischemic stroke (IS) is the most common subtype, accounting for 62.4% of all stroke cases (2). Globally, approximately 7.6 million new IS cases are diagnosed each year, with an age-standardized incidence rate of 98.62 per 100,000 person-years (3), highlighting an urgent need for improved prevention and clinical management.

Elevated serum uric acid (SUA) is significantly associated with the onset and progression of IS. Beyond physiological levels, SUA promotes inflammasome activation, increases proinflammatory cytokine release, and exacerbates oxidative stress and vascular inflammation, leading to endothelial dysfunction. These mechanisms collectively accelerate atherosclerosis and thrombus formation, thereby increasing stroke risk (4–6). Clinical studies have shown that SUA levels ≥ 340 μmol/L are associated with a 92.6% increase in recurrence risk among IS patients after adjusting for confounders (7). A meta-analysis further supports that hyperuricemia (HUA) is linked to a higher risk of IS recurrence (pooled OR = 1.80, 95% CI: 1.47–2.20) (8). Moreover, HUA has been identified as an independent predictor of adverse outcomes in patients with acute ischemic stroke (AIS) (9–13).

Beyond SUA, other metabolic abnormalities also contribute to the risk of IS. Impaired fasting glucose (IFG), which is defined as a fasting blood glucose (FBG) level of 6.1–6.9 mmol/L, is associated with an increased risk of IS (HR = 1.16, 95% CI: 1.07–1.25) (14). The triglyceride-glucose (TyG) index, a well-validated marker of insulin resistance, is also consistently linked to higher IS incidence in meta-analyses (15, 16). Interestingly, our previous study identified a dyslipidemia-rich phenotype among AIS patients via unsupervised clustering (17), reflecting lipid abnormalities in IS patients. Notably, HUA frequently coexists with disorders of glucose and lipid metabolism. There is a bidirectional relationship between HUA and hyperinsulinemia or diabetes mellitus (DM): impaired renal function secondary to dysglycemia reduces SUA excretion, while elevated SUA may aggravate glucose metabolic dysfunction (18). Consistently, Gu et al. reported that a higher TyG index, significantly increase the risk of incident HUA (19). HUA also commonly co-occurs with dyslipidemia, and its interaction synergistically promotes IS progression. Mechanistically, high SUA induces insulin resistance and disrupts lipid homeostasis (5, 20), both of which are central to the development of IS-related comorbidities such as diabetes and atherosclerosis.

Given that SUA, TyG, and lipid parameters are closely interlinked in the context of HUA and IS, a systematic evaluation of their combined role is essential for understanding underlying mechanisms and refining risk assessment. However, current evidence lacks a comprehensive assessment of these metabolic parameters, including SUA, TyG, and multiple lipid indices (e.g., TC, TG, LDL-C, HDL-C, non-HDL-C, atherogenic index of plasma [AIP], atherogenic coefficient [AC], lipoprotein combine index [LCI], Castelli’s risk index I [CRI-I], Castelli’s risk index II [CRI-II]) (21), as shared biomarkers in patients with comorbid HUA and IS. In recent years, as machine learning (ML) algorithms advance rapidly in healthcare, Professor Luo proposed the novel concept of Clinlabomics in 2022. This concept integrates multidimensional clinical and laboratory data with advanced ML algorithms to develop models for disease identification and risk assessment (22). Notably, Clinlabomics model can decipher the black-box nature of ML-derived models, enhance model transparency, and distinguish comorbid patients based on existing indicators. However, to date, there remains a lack of Clinlabomics models specifically dedicated to distinguishing HUA-IS comorbid patients.

To address this gap, we profiled these metabolic parameters in a comorbid cohort before and after 1:1 propensity score matching (PSM). We employed restricted cubic spline (RCS) analysis to explore potential non-linear associations, used 11 ML algorithms to develop Clinlabomics models, and applied the SHapley Additive exPlanations (SHAP) method to decipher the black-box nature of the optimal model and quantify the contributions of each indicator to the model’s estimates. This transparent and data-driven framework aims to facilitate the rapid identification of the HUA-IS comorbidity population.

## Materials and methods

### Study population

**Figure 1:**
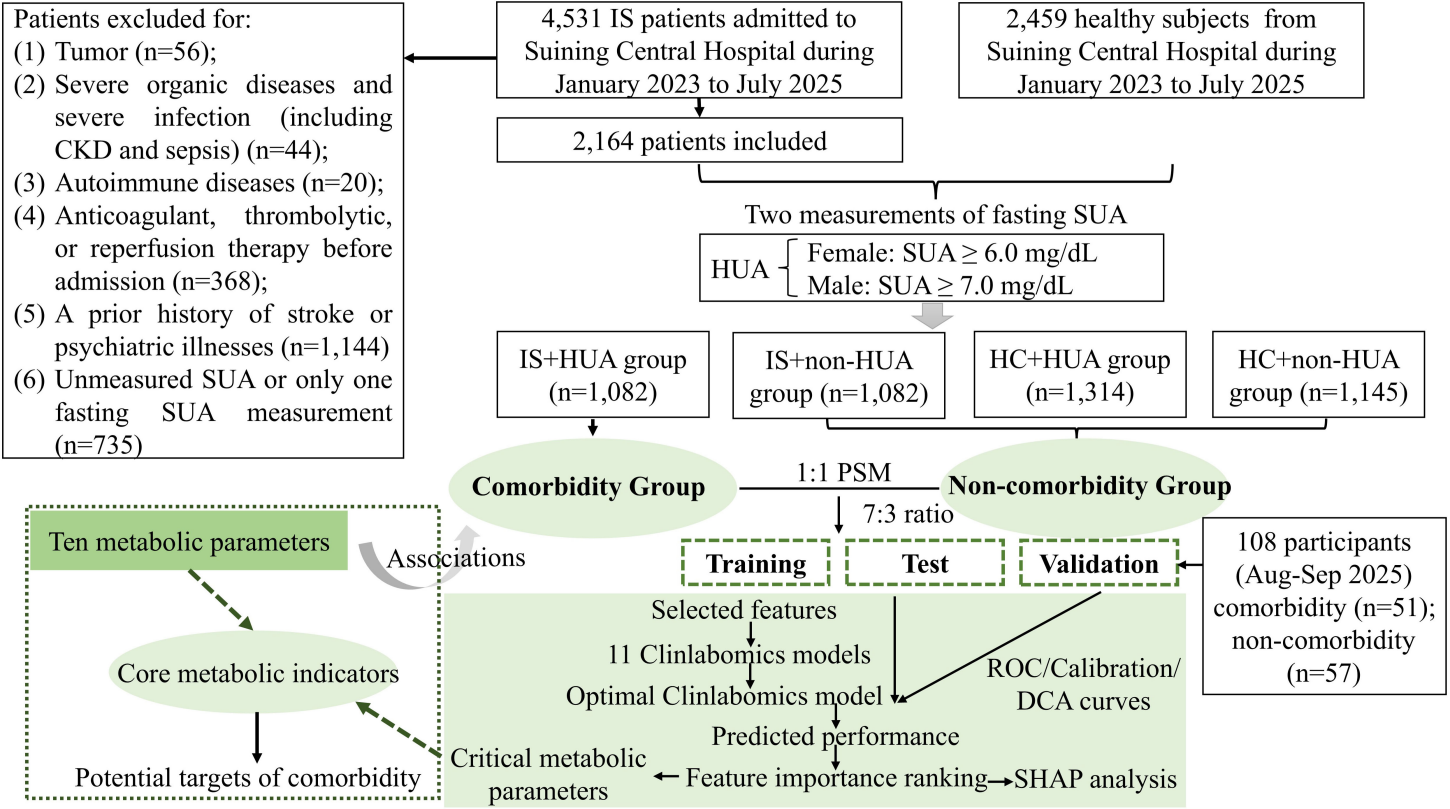
Flowchart of this study.

We retrospectively enrolled patients diagnosed with IS who were admitted to the Department of Neurology at Suining Central Hospital between January 2023 and July 2025. During hospitalization, all participants were required to have undergone at least two separate fasting SUA measurements on non-consecutive days, with the first conducted on the day of admission and the second on day 3 after admission. The inclusion criteria for IS patients were as follows: age ≥ 18 years and a first-time IS diagnosis confirmed by neuroimaging, such as cranial computed tomography (CT) or magnetic resonance imaging (MRI). Exclusion criteria included: tumor; chronic kidney disease (CKD) caused by diverse etiologies, severe dysfunction of major organs (heart, liver, kidney, or lungs); severe systemic infection or sepsis; autoimmune diseases; receipt of in-hospital anticoagulation, thrombolysis, or reperfusion therapy; a previous history of stroke or psychiatric disorders, or those who underwent only one SUA measurement after admission. Furthermore, we also concurrently included 2,459 relatively healthy subjects, defined as those who presented with IS-like symptoms but were confirmed non-IS via imaging examinations and had no tumors, CKD, severe organ function impairment, or infection. Based on prior studies (23, 24), HUA was defined as a SUA level ≥ 7.0 mg/dL (420 μmol/L) among males and ≥ 6.0 mg/dL (360 μmol/L) among females. This study was approved by the Ethics Committee of the Suining Central Hospital (No. KYLLKS20250126). Informed consent of all participants were involved. The flow diagram illustrating all analytical procedures in this study is presented in **Figure 1**.

### Data collection

We extracted general clinical characteristics of the participants from electronic medical records, including age, gender, marital status, ethnicity, height, weight, systolic blood pressure (SBP), diastolic blood pressure (DBP), and medical histories of DM, coronary heart disease (CHD), atrial fibrillation (AF), hypertension (HTN), hyperlipidemia (HLP); medication histories of antiplatelet therapy (APT), antihypertensive therapy, antidiabetic therapy, statin therapy, and urate-lowering therapy; as well as smoking and alcohol use. Hyperlipidemia (HLP) was defined as TG ≥ 150 mg/dl (1.70 mmol/L), TC ≥ 240 mg/dl (6.22 mmol/L), LDL-C ≥ 160 mg/dl (4.14 mmol/L), HDL-C < 40 mg/dl (1.04 mmol/L), or a medical diagnosis (25). All laboratory parameters, including routine blood tests, a coagulation profile, renal function tests, electrolytes, and a lipid panel, all of which were all measured on admission, were retrieved from the Laboratory Information System (LIS). Since the proportion of missing D-dimer values exceeded 60%, we did not incorporate it into the coagulation profile. Meanwhile, we also collected SUA of all participants on the 3rd day after admission. For patients with IS, we additionally collected the time from symptom onset to admission; Trial of Org 10172 in Acute Stroke Treatment (TOAST) classification; and modified Rankin Scale (mRS), National Institutes of Health Stroke Scale (NIHSS), and Glasgow Coma Scale (GCS) scores at admission. FBG was measured by the hexokinase method; UA was detected by the uricase-peroxidase method; TC was measured by the cholesterol oxidase method; TG was quantified by the glycerol-3-phosphate oxidase-peroxidase (GPO-POD) method; HDL-C and LDL-C were detected by direct methods, with all assays performed on a Beckman Coulter AU5800 analyzer. Besides, other relevant derived indices are provided below: neutrophil-to-lymphocyte ratio (NLR) = neutrophil (NEU)/lymphocyte (LYM), lymphocyte-to-monocyte ratio (LMR) = LYM/monocyte (MON), systemic inflammation index (SII) = platelet (PLT) * NEU/LYM (26), system inflammation response index (SIRI)=NEU*MON/LYM (27), platelet-to-neutrophil ratio (PNR) = PLT/NEU (28), platelet-to-lymphocyte ratio (PLR) = PLT/LYM (28), monocyte to high-density lipoprotein cholesterol ratio (MHR) = MON/high-density lipoprotein cholesterol (HDL-C) (29), neutrophil to high-density lipoprotein ratio (NHR) = NEU/HDL-C (30), platelet to high-density lipoprotein cholesterol ratio (PHR) = PLT/HDL-C (31), hemoglobin-to-red blood cell distribution width ratio (HRR) = hemoglobin (HGB)/red blood cell distribution width (RDW) (32), Hemoglobin, Albumin, Lymphocyte, Platelet Score (HALP) = hemoglobin (HGB) * albumin (ALB) * LYM/PLT (33), triglyceride-glucose index (TyG) = ln(fasting blood glucose [FBG] (mg/dL) * triglyceride [TG] (mg/dL)/2) (34), atherogenic index of plasma (AIP) = lg(TG/HDL-C) (35), atherogenic coefficient (AC) = non-high-density lipoprotein cholesterol (non-HDL-C)/HDL-C (36), lipoprotein combine index (LCI) = total cholesterol (TC) * TG * low-density lipoprotein cholesterol (LDL-C)/HDL-C (37, 38), Castelli’s risk index I (CRI-I) = TC/HDL-C (36), and Castelli’s risk index II (CRI-II) = LDL-C/HDL-C (36).

### Statistical analyses

For data with missing values below 5%, we performed multiple imputation using the “mice” package, with the number of iterations (parameter m) set to 5, which effectively handled the missing values. Normality of continuous variables was assessed using the Kolmogorov-Smirnov test. Variables conforming to a normal distribution were expressed as mean ± standard deviation (SD) and compared using the Student’s t-test; otherwise, they were summarized as median (Q1, Q3) and compared using the Mann-Whitney U test. Categorical variables were described as frequencies and percentages and compared by the chi-square test. To mitigate potential confounding effects, we performed 1:1 propensity score matching (PSM) (39), matching the comorbidity and non-comorbidity groups to 1,031 subjects. The covariates included age, gender, marriage, nationality, SBP, DBP, BMI, the bad habits of drinking and smoking; the histories of HTN, DM, AF and CHD, HLP; medication histories of APT, antihypertensive therapy, antidiabetic therapy, statin therapy, and urate-lowering therapy; and inflammatory markers (WBC count and SII). The propensity score was calculated for each participant using a caliper width of 0.02. Balance between groups after matching was evaluated with standardized mean difference (SMD) and quartile comparisons (40), using the Love plot for balance assessment. Post-matching results indicated well-balanced baseline characteristics between the two groups. Univariate and multivariate logistic regression were employed to assess the association between differential metabolic parameters and the risk of HUA-IS comorbidity before and after PSM. We categorized significant variables identified from the multivariate logistic regression analysis to quartile-based logistic regression. Three models were employed: Model 1 (crude model) included no covariates; Model 2 (partially adjusted model) was adjusted for demographic factors (age, gender, marital status, nationality, SBP, DBP, drinking status, smoking status, histories of HTN, DM, AF, CHD, HLP, and body mass index [BMI]); Model 3 (fully adjusted model) further incorporated medication histories of APT, antihypertensive therapy, antidiabetic therapy, statin therapy, urate-lowering therapy, and inflammatory status (WBC and SII). Subsequently, we performed restricted cubic spline (RCS) analysis on variables that remained significant in the quartile-based logistic regression to evaluate the dose-response relationship, with 4 knots placed at the 5th, 35th, 65th, and 95th percentiles. The matched dataset was split into training and internal validation sets in a 7:3 ratio. For variables with significant differences in the training set, we applied the LASSO method for regularization and coefficient shrinkage, thereby shrinking the coefficients of irrelevant features to zero and selecting features for subsequent model construction (41). In the training set, based on the selected features, 11 ML algorithms, including Generalized Linear Model (glm), Recursive Partitioning and Regression Trees (rpart), Naive Bayes (nb), Bayesian Generalized Linear Model (bayesglm), Random Forest (rf), eXtreme Gradient Boosting Tree (xgbTree), Support Vector Machine with Radial Basis Function Kernel (svmRadial), Support Vector Machine with Linear Kernel (svmLinear), Gradient Boosting Machine (gbm), Multivariate Adaptive Regression Splines (earth), and Elastic Net Regularized Regression (glmnet), were utilized to construct Clinlabomics models for HUA-IS comorbidity. Hyperparameter tuning was performed via grid search in the training set. Model performance was evaluated using the accuracy (ACC), F1-score, and area under the curve (AUC), with the optimal Clinlabomics model identified. Receiver operating characteristic (ROC) curves, calibration curve, and decision curve analysis (DCA) were used to evaluate the discriminative ability, the agreement between predicted probabilities and actual probabilities, and the clinical utility of the optimal model, respectively. Based on the optimal model, we also calculated its Brier score to further quantify the model’s calibration performance. Subsequently, we enrolled 108 participants from August to September 2025 as the independent validation cohort to evaluate the accuracy of the optimal Clinlabomics model. In addition, to interpret the contribution of individual features to the model, the SHAP algorithm was applied to compute SHAP values from the best-performing Clinlabomics model (42). SHAP values quantify the marginal contribution of each feature to the estimated outcome. This approach supports both local and global interpretability of the model. The entire process for Clinlabomics model development was performed using the “Icare” package (https://github.com/OmicsLY/Icare). All statistical analyses were performed using RStudio (Version 4.3.0). A two-sided *P*-value < 0.05 was considered statistically significant.

## Results

### Baseline characteristics of participants

A total of 2,164 IS patients and 2,459 healthy controls (HCs) were included in this study. Based on two SUA levels, subjects were stratified into four groups: non-HUA HCs (n=1,145), HUA HCs (1,314), non-HUA IS (n=1,082), and HUA-IS comorbidity (n=1,082) group, with differences in characteristics across groups presented in **Table 1**. A detailed flowchart is provided in **Figure 1**. **Supplementary Table S1** presents missing data proportions and data distributions before and after imputation, with no significant differences detected in the latter. Meanwhile, we compared the differences of indicators between non-HUA IS patients and non-HUA HCs (**Supplementary Table S2**), as well as between HUA HCs and non-HUA HCs (**Supplementary Table S3**). Through these comparisons, we identified differentially expressed indicators closely associated with IS and HUA, respectively. To further investigate the potential pathogenic mechanism of the comorbidity group, we merged all groups except the HUA-IS comorbidity group into a non-comorbidity group and compared the differences in indicators (**Supplementary Table S4**). Subsequently, we took the intersection of the differentially expressed indicators from these three groups (**Supplementary Table S5**), identifying 33 indicators closely associated with IS, HUA, and comorbidity (**Supplementary Figure 1**). Among these, ten metabolic indicators, including UA_admission, UA_3d, TyG, TG, HDL-C, AIP, AC, LCI, CRI-I, and CRI-II, were found to be closely correlated with the comorbidity, indicating no significant biological confounding. After PSM, both the comorbidity and non-comorbidity groups included 1,031 cases each, with 2,561 participants (55.4%). The standardized mean difference (SMD) values of these covariates were all less than 0.1, as visualized via a Love plot (**Supplementary Table S6**, **Supplementary Figure 2**). Significant differences (all *P* < 0.05) were observed between the two groups in nationality, DBP, MON, NLR, LMR, SII, SIRI, PLR, MHR, HRR, HALP, RBC, HGB, HCT, RDW-CV, TG, AIP, LCI, TyG, UREA, CREA, UA_admission, UA_3d, and TT (**Table 2**). Additionally, the dataset was randomly split into a training set and a testing set at a 7:3 ratio, with intergroup differences in the training set presented in **Table 3**. Furthermore, the detailed characteristics of all clinical and laboratory results in the independent validation cohort were presented in **Table 4**.

**Table 1:** Main characteristics of included participants before PSM.

Variables	Total (n = 4,623)	Non-HUA HCs (n = 1,145)	HUA HCs (n = 1,314)	Non-HUA IS (n = 1,082)	Comorbidity (n = 1,082)	*P*
Age (years)	67 (56, 75)	64 (56, 72)	61 (49, 72)	71 (60, 78)	71 (60, 78)	**< 0.001**
Gender (Male, n, %)	2655 (57)	494 (43)	810 (62)	675 (62)	676 (62)	**< 0.001**
Marriage (Other status, n, %)	456 (10)	86 (8)	146 (11)	116 (11)	108 (10)	**0.016**
Nationality (Ethnic minority, n, %)	171 (4)	92 (8)	76 (6)	1 (0)	2 (0)	**< 0.001**
APT, n (Yes, %)	430 (9)	10 (1)	13 (1)	182 (17)	225 (21)	**< 0.001**
Antihypertensive therapy, n (Yes, %)	1770 (38)	165 (14)	240 (18)	642 (59)	723 (67)	**< 0.001**
Antidiabetic therapy, n (Yes, %)	592 (13)	34 (3)	63 (5)	241 (22)	254 (23)	**< 0.001**
Statins therapy, n (Yes, %)	435 (9)	8 (1)	13 (1)	182 (17)	232 (21)	**< 0.001**
Urate-lowering therapy, n (Yes, %)	64 (1)	0 (0)	25 (2)	0 (0)	39 (4)	**< 0.001**
Time of onset (h)	48 (13.2, 168)	-	-	48 (12, 144)	48 (15, 168)	–
TOAST, n (%)						–
LAA	683 (15)	-	-	358 (33)	325 (30)	
SAO	1182 (26)	-	-	580 (54)	602 (56)	
CE	163 (4)	-	-	74 (7)	89 (8)	
SOE	92 (2)	-	-	45 (4)	47 (4)	
SUE	44 (1)	-	-	25 (2)	19 (2)	
GCS_admission	15 (15,15)	-	-	1 (0,1)	1 (0,1)	–
mRS_admission	1 (0,1)	-	-	15 (15, 15)	15 (15, 15)	–
NIHSS_admission	2 (1,4)	-	-	2 (1, 4)	1 (0, 3)	–
SBP (mmHg)	137 (122, 151)	132 (119, 143)	130 (119, 145)	141 (128, 157)	139 (125, 157)	**< 0.001**
DBP (mmHg)	83 (73, 90)	80 (73, 87)	82 (72, 90)	83 (75, 94)	83 (74, 94)	**< 0.001**
Drinking (Yes, n, %)	743 (16)	98 (9)	178 (14)	224 (21)	243 (22)	**< 0.001**
Smoking (Yes, n, %)	443 (10)	75 (7)	116 (9)	128 (12)	124 (11)	**< 0.001**
HTN (Yes, n, %)	2703 (58)	495 (43)	682 (52)	731 (68)	795 (73)	**< 0.001**
DM (Yes, n, %)	870 (19)	141 (12)	168 (13)	273 (25)	288 (27)	**< 0.001**
AF (Yes, n, %)	117 (3)	4 (0)	16 (1)	40 (4)	57 (5)	**< 0.001**
CHD (Yes, n, %)	277 (6)	36 (3)	86 (7)	69 (6)	86 (8)	**< 0.001**
HLP (Yes, n, %)	2534 (55)	510 (45)	774 (59)	572 (53)	678 (63)	**< 0.001**
BMI (Kg/m^2)	25.14 (23.01, 27.1)	23.88 (22.22, 26.03)	24.77 (22.72, 27.28)	25.86 (23.97, 27.30)	25.81 (24.03, 27.47)	**< 0.001**
WBC (10^9/L)	6.7 (5.5, 8.2)	6.2 (5.1, 7.6)	6.5 (5.5, 7.9)	7.0 (5.7, 8.6)	7.1 (5.8, 9.0)	**< 0.001**
NEU (10^9/L)	4.41 (3.34, 5.94)	3.98 (2.92, 5.46)	4.08 (3.21, 5.60)	4.80 (3.65, 6.49)	4.82 (3.71, 6.46)	**< 0.001**
LYM (10^9/L)	1.46 (1.08, 1.86)	1.47 (1.08, 1.87)	1.55 (1.17, 1.97)	1.37 (1.03, 1.72)	1.41 (1.03, 1.86)	**< 0.001**
MON (10^9/L)	0.51 (0.38, 0.83)	0.42 (0.34, 0.54)	0.45 (0.36, 0.56)	0.81 (0.47, 1.41)	0.9 (0.47, 1.54)	**< 0.001**
NLR	2.96 (2.01, 4.69)	2.55 (1.74, 4.50)	2.63 (1.75, 3.98)	3.48 (2.37, 5.46)	3.27 (2.33, 5.19)	**< 0.001**
LMR	2.65 (1.00, 4.02)	3.42 (2.36, 4.61)	3.50 (2.50, 4.57)	1.00 (1.00, 2.89)	1.00 (1.00, 2.77)	**< 0.001**
SII	550 (356, 907)	488 (305, 829)	480 (312, 794)	665 (426, 1059)	612 (395, 969)	**< 0.001**
SIRI	1.86 (0.91, 3.92)	1.12 (0.67, 2.19)	1.20 (0.74, 2.02)	3.58 (1.72, 5.45)	3.59 (1.70, 5.44)	**< 0.001**
PNR	41.79 (29.56, 58.09)	47.12 (32.17, 65.43)	44.88 (32.04, 61.78)	39.22 (27.62, 52.63)	38.69 (27.2, 50.80)	**< 0.001**
PLR	129.19 (95.94, 171.48)	125.47 (93.02, 169.47)	119.38 (93.12, 153.10)	140.40 (105.51, 189.4)	130.76 (95.38, 178.25)	**< 0.001**
MHR	0.43 (0.29, 0.71)	0.34 (0.24, 0.46)	0.36 (0.27, 0.49)	0.64 (0.39, 1.12)	0.71 (0.39, 1.26)	**< 0.001**
NHR	3.53 (2.56, 4.98)	3.08 (2.17, 4.34)	3.34 (2.51, 4.62)	3.91 (2.78, 5.57)	3.96 (2.93, 5.53)	**< 0.001**
PHR	146.43 (110.70, 193.17)	138.33 (103.60, 188.19)	147.87 (115.82, 189.74)	148.6 (111.89, 196.69)	148.44 (112.31, 198.86)	**< 0.001**
HRR	9.92 (8.59, 11.03)	9.70 (8.48, 10.92)	10.32 (8.96, 11.37)	9.65 (8.48, 10.68)	9.78 (8.42, 10.91)	**< 0.001**
HALP	41.55 (28.60, 57.09)	41.09 (28.60, 56.95)	46.48 (33.45, 61.67)	36.85 (24.65, 51.21)	41.56 (27.87, 56.69)	**< 0.001**
RBC (10^12/L)	4.33 (3.90, 4.77)	4.25 (3.80, 4.65)	4.43 (4.00, 4.90)	4.26 (3.89, 4.68)	4.36 (3.93, 4.79)	**< 0.001**
HGB (g/L)	132 (118, 144)	128 (115, 141)	137 (120, 148)	130 (117, 141)	132 (118, 145)	**< 0.001**
HCT (%)	39.7 (35.9, 43.5)	39.0 (35.3, 42.7)	41.0 (36.3, 44.8)	39.2 (35.6, 42.6)	39.85 (36.2, 43.8)	**< 0.001**
MCV (fL)	92.4 (89.2, 95.5)	93.1 (89.9, 96.0)	92.2 (88.9, 95.4)	92.2 (88.7, 95.3)	92.1 (89.1, 95.2)	**< 0.001**
MCHC (g/L)	331 (324, 337)	330 (323, 336)	331 (324, 337)	331 (324, 338)	331 (324, 338)	**0.002**
MCH (pg)	30.6 (29.4, 31.8)	30.7 (29.6, 31.9)	30.4 (29.3, 31.7)	30.6 (29.4, 31.7)	30.5 (29.3, 31.7)	**0.024**
RDW-CV (%)	13.3 (12.8, 14.1)	13.2 (12.6, 13.9)	13.2 (12.6, 13.9)	13.4 (12.9, 14.2)	13.6 (13.0, 14.3)	**< 0.001**
PLT (10^9/L)	186 (146, 227)	183 (145, 226)	183 (147, 218)	189 (149, 229)	187 (143, 232)	**0.038**
CRP (mg/L)	3.70 (1.03, 6.46)	3.70 (1.11, 6.04)	3.70 (1.05, 6.01)	3.31 (0.92, 14.24)	3.43 (1.01, 14.24)	**< 0.001**
TC (mmol/L)	4.46 (3.71, 5.24)	4.44 (3.80, 5.14)	4.52 (3.79, 5.21)	4.38 (3.59, 5.32)	4.46 (3.64, 5.34)	0.154
TG (mmol/L)	1.45 (1.00, 2.13)	1.24 (0.94, 1.77)	1.56 (1.08, 2.36)	1.36 (0.96, 1.98)	1.64 (1.11, 2.38)	**< 0.001**
LDL-C (mmol/L)	2.68 (2.15, 3.27)	2.63 (2.21, 3.13)	2.72 (2.19, 3.31)	2.69 (2.03, 3.33)	2.73 (2.09, 3.35)	0.278
HDL-C (mmol/L)	1.25 (1.05, 1.48)	1.29 (1.08, 1.54)	1.22 (1.05, 1.46)	1.25 (1.05, 1.47)	1.22 (1.04, 1.45)	**< 0.001**
Non-HDL-C (mmol/L)	3.16 (2.50, 3.89)	3.10 (2.53, 3.72)	3.26 (2.55, 3.91)	3.14 (2.39, 3.98)	3.20 (2.45, 4.01)	**0.003**
AIP	0.06 (-0.11, 0.27)	0 (-0.17, 0.17)	0.10 (-0.06, 0.30)	0.05 (-0.14, 0.23)	0.12 (-0.06, 0.34)	**< 0.001**
AC	2.57 (1.95, 3.23)	2.37 (1.85, 2.98)	2.66 (2.03, 3.28)	2.52 (1.90, 3.25)	2.61 (1.99, 3.33)	**< 0.001**
LCI	13.96 (7.41, 25.87)	11.27 (6.90, 19.20)	16.38 (8.50, 29.25)	12.98 (6.62, 24.05)	16.16 (8.00, 31.89)	**< 0.001**
CRI-I	3.57 (2.95, 4.23)	3.37 (2.85, 3.98)	3.66 (3.03, 4.28)	3.52 (2.90, 4.25)	3.61 (2.99, 4.33)	**< 0.001**
CRI-II	2.16 (1.68, 2.69)	2.03 (1.62, 2.55)	2.28 (1.71, 2.71)	2.14 (1.67, 2.75)	2.22 (1.70, 2.77)	**< 0.001**
FBG (mmol/L)	5.84 (5.01, 7.52)	5.48 (4.89, 6.54)	5.43 (4.82, 6.52)	6.51 (5.32, 8.56)	6.40 (5.31, 8.43)	**< 0.001**
TyG	8.86 (8.43, 9.36)	8.66 (8.26, 9.07)	8.86 (8.47, 9.36)	8.91 (8.45, 9.41)	9.08 (8.61, 9.62)	**< 0.001**
UREA (mmol/L)	6.19 (4.96, 7.84)	5.97 (4.87, 7.15)	6.26 (5.00, 8.20)	5.81 (4.66, 7.34)	6.86 (5.44, 9.03)	**< 0.001**
CREA (μmol/L)	71.5 (59.8, 88.5)	62.0 (52.1, 71.5)	75.0 (63.6, 92.9)	69.0 (58.0, 83.7)	85.9 (69.7, 110.6)	**< 0.001**
UA_admission (μmol/L)	382 (288, 457)	278 (232, 321)	453 (421, 495)	293 (238, 342)	458 (425, 514)	**< 0.001**
UA_3d (μmol/L)	366 (264, 466)	248 (195, 306)	454 (398, 515)	270 (212, 333)	464 (413, 513)	**< 0.001**
K (mmol/L)	3.86 (3.62, 4.12)	3.87 (3.66, 4.09)	3.89 (3.65, 4.17)	3.81 (3.56, 4.05)	3.87 (3.58, 4.17)	**< 0.001**
Na (mmol/L)	140.6 (138.9, 142.1)	140.9 (139.2, 142.2)	140.8 (139.1, 142.0)	140.2 (138.2, 141.9)	140.4 (138.6, 142.1)	**< 0.001**
Cl (mmol/L)	105.0 (103.0, 107.0)	105.6 (104.0, 107.4)	105.1 (103.3, 107.3)	104.5 (102.3, 106.5)	104.4 (102.0, 106.6)	**< 0.001**
PTA (%)	112 (99, 126)	111 (100, 124)	112 (100, 126)	112 (98, 126)	113 (98, 127)	0.775
TT (s)	16.8 (15.6, 17.8)	16.3 (15.1, 17.4)	16.7 (15.4, 17.7)	17.1 (15.9, 18.2)	17.1 (16.0, 18.2)	**< 0.001**
INR	0.98 (0.93, 1.03)	0.98 (0.94, 1.03)	0.98 (0.93, 1.03)	0.97 (0.92, 1.03)	0.97 (0.92, 1.03)	**< 0.001**
APTT (s)	28.2 (26.4, 30.3)	27.9 (26.3, 30.1)	28.2 (26.3, 30.4)	28.3 (26.7, 30.4)	28.3 (26.6, 30.3)	**0.006**
PT (s)	11.0 (10.5, 11.6)	11.0 (10.6, 11.5)	11.0 (10.5, 11.4)	11.1 (10.5, 11.9)	11.1 (10.6, 11.8)	**< 0.001**
FIB (g/L)	2.92 (2.46, 3.59)	2.84 (2.41, 3.43)	2.75 (2.32, 3.34)	3.12 (2.58, 3.82)	3.12 (2.55, 3.83)	**< 0.001**

HUA, hyperuricemia; IS, ischemic stroke; PSM, propensity score matching; APT, antiplatelet therapy; SBP, systolic blood pressure; DBP, diastolic blood pressure; TOAST, Trial of Org 10172 in Acute Stroke Treatment; LAA, large-artery atherosclerosis; SAO, small artery occlusion; CE, Cardio embolism; SOE, stroke of other determined etiologies; SUE, stroke of undetermined etiologies; GCS, Glasgow Coma Scale; mRS, Modified Rankin Scale; NIHSS, National Institutes of Health Stroke Scale; HTN, hypertension; AF, atrial fibrillation; CHD, coronary heart disease; HLP, hyperlipidemia; DM, diabetes mellitus; BMI, body mass index; NLR, neutrophil-to-lymphocyte ratio; LMR, lymphocyte-to-monocyte ratio; SII, systemic inflammatory index; PLR, platelet-to-lymphocyte ratio; HALP, hemoglobin, albumin, lymphocyte, platelet score; RBC, red blood cell; HGB, hemoglobin; HCT, hematocrit; MCHC, mean corpuscular hemoglobin concentration; RDW-CV, red blood cell distribution width-coefficient of variation; TC, total cholesterol; TG, triglyceride; LDL-C, low-density lipoprotein cholesterol; HDL-C, high-density lipoprotein cholesterol; non-HDL-C, non-high-density lipoprotein cholesterol; AIP, atherogenic index of plasma; AC, atherogenic coefficient; LCI, lipoprotein combine index; CRI-I, Castelli’s index-I; CRI-II, Castelli’s index-II; FBG, fasting blood glucose; TyG, triglyceride-glucose index; K, potassium; Na, sodium; UA, uric acid; PTA, prothrombin activity; TT, thrombin time; INR, international normalized ratio; APTT, activated partial thromboplastin time; PT, prothrombin time; FIB, fibrinogen. Bold font indicates statistically significant differences.

**Table 2:** Basic characteristics of included participants stratified by comorbidity following PSM.

Variables	Non-comorbidity (N = 1,031)	Comorbidity (N = 1,031)	*P*
Age (years)	71 (60, 78)	70 (59, 78)	0.500
Gender (Male, n, %)	618 (60)	632 (61)	0.558
Marriage (Other status, n, %)	99 (10)	103 (10)	0.824
Nationality (Ethnic minority, n, %)	18 (2)	2 (0)	< 0.001
APT, n (Yes, %)	165 (16)	188 (18)	0.198
Antihypertensive therapy, n (Yes, %)	688 (67)	674 (65)	0.545
Antidiabetic therapy, n (Yes, %)	231 (22)	236 (23)	0.833
Statins therapy, n (Yes, %)	178 (17)	195 (19)	0.360
Urate-lowering therapy, n (Yes, %)	20 (2)	23 (2)	0.758
Time of onset (h)	48 (13, 168)	48 (15, 168)	-
TOAST, n (%)			-
LAA	217 (21)	306 (30)	
SAO	324 (31)	582 (56)	
CE	43 (4)	81 (8)	
SOE	22 (2)	44 (4)	
SUE	16 (2)	18 (2)	
GCS_admission	15 (15, 15)	15 (15, 15)	-
mRS_admission	1 (0, 2)	1 (0, 1)	-
NIHSS_admission	2 (0, 4)	1 (0, 3)	-
SBP (mmHg)	138 (125, 155)	139 (125, 157)	0.473
DBP (mmHg)	83 (74, 91)	83 (74, 94)	0.015
Drinking (Yes, n, %)	218 (21)	225 (22)	0.748
Smoking (Yes, n, %)	104 (10)	114 (11)	0.519
HTN (Yes, n, %)	754 (73)	750 (73)	0.882
DM (Yes, n, %)	268 (26)	271 (26)	0.920
AF (Yes, n, %)	36 (3)	50 (5)	0.152
CHD (Yes, n, %)	77 (7)	81 (8)	0.804
HLP (Yes, n, %)	606 (59)	640 (62)	0.137
BMI (Kg/m^2)	25.73 (23.84, 27.41)	25.78 (23.94, 27.41)	0.847
WBC (10^9/L)	7.2 (5.9, 8.6)	7.0 (5.7, 8.9)	0.505
NEU (10^9/L)	4.93 (3.78, 6.57)	4.79 (3.68, 6.42)	0.271
LYM (10^9/L)	1.37 (1.02, 1.77)	1.41 (1.04, 1.86)	0.063
MON (10^9/L)	0.57 (0.41, 0.98)	0.91 (0.48, 1.56)	< 0.001
NLR	3.55 (2.37, 5.70)	3.23 (2.32, 5.11)	0.026
LMR	2.22 (1.00, 3.46)	1.00 (1.00, 2.78)	< 0.001
SII	661 (419, 1065)	606 (393, 951)	0.017
SIRI	2.57 (1.22, 4.70)	3.59 (1.67, 5.40)	< 0.001
PNR	38.11 (26.55, 52.23)	38.87 (27.31, 51.08)	0.850
PLR	137.67 (101.25, 184.63)	130.12 (95.39, 176.1)	0.020
MHR	0.49 (0.33, 0.82)	0.72 (0.39, 1.26)	< 0.001
NHR	4.05 (2.95, 5.62)	3.92 (2.9, 5.44)	0.424
PHR	150.28 (111.18, 197.71)	147.90 (111.49, 197.13)	0.662
HRR	9.59 (8.48, 10.68)	9.85 (8.48, 10.92)	0.020
HALP	37.31 (25.94, 53.17)	41.98 (28.2, 56.79)	< 0.001
RBC (10^12/L)	4.24 (3.89, 4.68)	4.37 (3.94, 4.80)	< 0.001
HGB (g/L)	129 (117, 141)	133 (119, 145)	< 0.001
HCT (%)	39.0 (35.4, 42.6)	39.9 (36.2, 43.9)	< 0.001
MCV (fL)	92.1 (88.8, 95.1)	92.1 (89.1, 95.3)	0.668
MCHC (g/L)	331 (324, 337)	331 (324, 338)	0.873
MCH (pg)	30.5 (29.3, 31.7)	30.5 (29.4, 31.7)	0.958
RDW-CV (%)	13.4 (12.9, 14.2)	13.5 (13.0, 14.3)	0.031
PLT (10^9/L)	188 (147, 229)	186 (143, 229)	0.673
CRP (mg/L)	4.06 (1.19, 9.98)	3.22 (1.00, 14.24)	0.379
TC (mmol/L)	4.39 (3.61, 5.20)	4.48 (3.66, 5.34)	0.138
TG (mmol/L)	1.43 (0.99, 2.08)	1.64 (1.12, 2.38)	< 0.001
LDL-C (mmol/L)	2.63 (2.05, 3.25)	2.73 (2.10, 3.36)	0.090
HDL-C (mmol/L)	1.23 (1.02, 1.44)	1.22 (1.04, 1.46)	0.916
Non-HDL-C (mmol/L)	3.09 (2.44, 3.88)	3.21 (2.46, 4.01)	0.107
AIP	0.07 (-0.11, 0.26)	0.12 (-0.06, 0.34)	< 0.001
AC	2.56 (1.96, 3.25)	2.60 (1.99, 3.33)	0.178
LCI	13.55 (7.46, 24.64)	16.16 (8.03, 31.48)	< 0.001
CRI-I	3.56 (2.96, 4.25)	3.60 (2.99, 4.33)	0.178
CRI-II	2.14 (1.70, 2.74)	2.21 (1.70, 2.75)	0.158
FBG (mmol/L)	6.26 (5.22, 8.29)	6.41 (5.31, 8.39)	0.308
TyG	8.94 (8.51, 9.42)	9.08 (8.63, 9.61)	< 0.001
UREA (mmol/L)	6.05 (4.90, 7.49)	6.82 (5.41, 8.96)	< 0.001
CREA (μmol/L)	69.6 (58.9, 85.8)	84.1 (69.3, 107.6)	< 0.001
UA_admission (μmol/L)	312 (250, 379)	456 (424, 512)	< 0.001
UA_3d (μmol/L)	290 (220, 366)	463 (412, 514)	< 0.001
K (mmol/L)	3.84 (3.60, 4.08)	3.86 (3.58, 4.16)	0.154
Na (mmol/L)	140.3 (138.5, 142.1)	140.4 (138.7, 142.1)	0.487
Cl (mmol/L)	104.7 (102.4, 106.9)	104.4 (102.1, 106.6)	0.067
PTA (%)	112 (98, 126)	113 (99, 127)	0.369
TT (s)	16.9 (15.8, 17.9)	17.2 (16.1, 18.2)	< 0.001
INR	0.97 (0.93, 1.04)	0.97 (0.92, 1.03)	0.113
APTT (s)	28.0 (26.3, 30.0)	28.2 (26.5, 30.2)	0.090
PT (s)	11.1 (10.6, 11.8)	11.1 (10.6, 11.7)	0.651
FIB (g/L)	3.02 (2.54, 3.80)	3.12 (2.54, 3.81)	0.352

APT, antiplatelet therapy; SBP, systolic blood pressure; DBP, diastolic blood pressure; TOAST, Trial of Org 10172 in Acute Stroke Treatment; LAA, large-artery atherosclerosis; SAO, small artery occlusion; CE, Cardioembolism; SOE, stroke of other determined etiologies; SUE, stroke of undetermined etiologies; GCS, Glasgow Coma Scale; mRS, Modified Rankin Scale; NIHSS, National Institutes of Health Stroke Scale; HTN, hypertension; AF, atrial fibrillation; CHD, coronary heart disease; HLP, hyperlipidemia; DM, diabetes mellitus; BMI, body mass index; NLR, neutrophil-to-lymphocyte ratio; LMR, lymphocyte-to-monocyte ratio; SII, systemic inflammatory index; PLR, platelet-to-lymphocyte ratio; HALP, hemoglobin, albumin, lymphocyte, platelet score; RBC, red blood cell; HGB, hemoglobin; HCT, hematocrit; MCHC, mean corpuscular hemoglobin concentration; RDW-CV, red blood cell distribution width-coefficient of variation; TC, total cholesterol; TG, triglyceride; LDL-C, low-density lipoprotein cholesterol; HDL-C, high-density lipoprotein cholesterol; non-HDL-C, non-high-density lipoprotein cholesterol; AIP, atherogenic index of plasma; AC, atherogenic coefficient; LCI, lipoprotein combine index; CRI-I, Castelli’s index-I; CRI-II, Castelli’s index-II; FBG, fasting blood glucose; TyG, triglyceride-glucose index; K, potassium; Na, sodium; UA, uric acid; PTA, prothrombin activity; TT, thrombin time; INR, international normalized ratio; APTT, activated partial thromboplastin time; PT, prothrombin time; FIB, fibrinogen.

**Table 3:** The characteristics of included participants classified by comorbidity in training dataset.

Variables	Non-comorbidity (N = 722)	Comorbidity (N = 722)	*P*
Age (years)	71 (61, 78)	70 (59, 78)	0.207
Gender (Male, n, %)	440 (61)	437 (61)	0.914
Marriage (Other status, n, %)	76 (11)	77 (11)	1
Nationality (Ethnic minority, n, %)	16 (2.2)	0 (0)	**< 0.001**
APT, n (Yes, %)	111 (15)	140 (19)	**0.044**
Antihypertensive therapy, n (Yes, %)	477 (66)	462 (64)	0.440
Antidiabetic therapy, n (Yes, %)	157 (22)	161 (22)	0.849
Statins therapy, n (Yes, %)	121 (17)	142 (20)	0.173
Urate-lowering therapy, n (Yes, %)	14 (2)	13 (2)	1
Time of onset (h)	48 (12, 168)	48 (15, 168)	
TOAST, n (%)			-
LAA	149 (21)	216 (30)	
SAO	222 (31)	407 (56)	
CE	32 (4)	56 (8)	
SOE	17 (2)	30 (4)	
SUE	14 (2)	13 (2)	
GCS_admission	15 (15, 15)	15 (15, 15)	-
mRS_admission	1 (0, 1.25)	1 (0, 1)	-
NIHSS_admission	2 (0, 4)	1 (0, 3)	-
SBP (mmHg)	139 (125, 155)	138 (124, 156)	0.501
DBP (mmHg)	83 (74, 91)	83 (74, 93)	0.355
Drinking (Yes, n, %)	151 (21)	165 (23)	0.408
Smoking (Yes, n, %)	73 (10)	79 (11)	0.668
HTN (Yes, n, %)	525 (73)	515 (71)	0.598
DM (Yes, n, %)	178 (25)	190 (26)	0.507
AF (Yes, n, %)	27 (4)	36 (5)	0.303
CHD (Yes, n, %)	49 (7)	58 (8)	0.422
HLP (Yes, n, %)	429 (59)	439 (61)	0.629
BMI (Kg/m^2)	25.63 (23.81, 27.39)	25.8 (24.06, 27.53)	0.280
WBC (10^9/L)	7.1 (5.8, 8.7)	7.0 (5.7, 8.9)	0.797
NEU (10^9/L)	4.90 (3.69, 6.71)	4.75 (3.71, 6.40)	0.394
LYM (10^9/L)	1.35 (0.98, 1.73)	1.44 (1.04, 1.87)	**0.005**
MON (10^9/L)	0.57 (0.40, 1.00)	0.90 (0.47, 1.54)	**< 0.001**
NLR	3.56 (2.42, 5.84)	3.20 (2.28, 5.04)	**0.009**
LMR	2.18 (1.00, 3.35)	1.00 (1.00, 2.81)	**< 0.001**
SII	684 (410, 1,095)	599 (387, 932)	**0.014**
SIRI	2.72 (1.24, 4.68)	3.58 (1.65, 5.30)	**< 0.001**
PNR	37.61 (25.94, 52.48)	38.89 (27.62, 51.04)	0.804
PLR	138.66 (101.66, 186.68)	128.98 (94.32, 173.3)	**0.007**
MHR	0.49 (0.32, 0.82)	0.72 (0.39, 1.21)	**< 0.001**
NHR	4.04 (2.91, 5.64)	3.94 (2.88, 5.35)	0.469
PHR	147.64 (110.11, 197.69)	148.42 (109.72, 197.5)	0.910
HRR	9.58 (8.52, 10.68)	9.77 (8.43, 11.00)	0.080
HALP	36.83 (25.55, 51.97)	42.3 (28.65, 57.35)	**< 0.001**
RBC (10^12/L)	4.24 (3.88, 4.69)	4.36 (3.91, 4.80)	**0.005**
HGB (g/L)	129 (117, 142)	132 (118, 145)	**0.005**
HCT (%)	39.1 (35.4, 42.6)	39.9 (36.2, 43.9)	**0.003**
MCV (fL)	92.2 (88.7, 95.4)	92.1 (88.8, 95.6)	0.744
MCHC (g/L)	331 (324, 338)	330 (324, 338)	0.715
MCH (pg)	30.60 (29.30, 31.70)	30.50 (29.30, 31.80)	0.849
RDW-CV (%)	13.5 (12.9, 14.2)	13.6 (12.9, 14.2)	0.213
PLT (10^9/L)	186 (144, 226)	187 (144, 232)	0.697
CRP (mg/L)	4.12 (1.15, 9.94)	2.86 (0.89, 14.24)	0.102
TC (mmol/L)	4.43 (3.61, 5.20)	4.48 (3.71, 5.33)	0.257
TG (mmol/L)	1.42 (0.98, 2.07)	1.61 (1.11, 2.38)	**< 0.001**
LDL-C (mmol/L)	2.65 (2.05, 3.28)	2.72 (2.11, 3.37)	0.390
HDL-C (mmol/L)	1.24 (1.01, 1.45)	1.23 (1.05, 1.48)	0.733
Non-HDL-C (mmol/L)	3.15 (2.46, 3.87)	3.20 (2.51, 4.02)	0.209
AIP	0.06 (-0.11, 0.25)	0.11 (-0.07, 0.34)	**< 0.001**
AC	2.56 (1.94, 3.28)	2.60 (1.99, 3.33)	0.292
LCI	14.03 (7.39, 24.85)	15.77 (7.69, 32.16)	**0.001**
CRI-I	3.56 (2.94, 4.28)	3.60 (2.99, 4.33)	0.292
CRI-II	2.14 (1.69, 2.77)	2.22 (1.69, 2.73)	0.492
FBG (mmol/L)	6.34 (5.22, 8.38)	6.32 (5.29, 8.26)	0.896
TyG	8.94 (8.51, 9.42)	9.04 (8.60, 9.56)	**0.003**
UREA (mmol/L)	5.98 (4.92, 7.49)	6.88 (5.45, 9.02)	**< 0.001**
CREA (μmol/L)	69.4 (58.4, 85.1)	83.0 (69.1, 105.4)	**< 0.001**
UA_admission (μmol/L)	308 (248, 373)	456 (423, 511)	**< 0.001**
UA_3d (μmol/L)	286 (219, 363)	462 (411, 513)	**< 0.001**
K (mmol/L)	3.84 (3.61, 4.08)	3.86 (3.58, 4.16)	0.227
Na (mmol/L)	140.3 (138.4, 142.0)	140.5 (138.8, 142.2)	0.240
Cl (mmol/L)	104.7 (102.4, 107.0)	104.4 (102.0, 106.7)	0.120
PTA (%)	112 (98, 126)	113 (99, 127)	0.241
TT (s)	16.9 (15.7, 17.9)	17.2 (16.2, 18.2)	**< 0.001**
INR	0.97 (0.93, 1.04)	0.97 (0.92, 1.02)	0.067
APTT (s)	28.0 (26.3, 29.9)	28.3 (26.6, 30.2)	0.097
PT (s)	11.1 (10.5, 11.9)	11.1 (10.6, 11.8)	0.825
FIB (g/L)	3.00 (2.51, 3.76)	3.06 (2.52, 3.76)	0.565

SBP, systolic blood pressure; DBP, diastolic blood pressure; TOAST, Trial of Org 10172 in Acute Stroke Treatment; LAA, large-artery atherosclerosis; SAO, small artery occlusion; CE, Cardioembolism; SOE, stroke of other determined etiologies; SUE, stroke of undetermined etiologies; GCS, Glasgow Coma Scale; mRS, Modified Rankin Scale; NIHSS, National Institutes of Health Stroke Scale; HTN, hypertension; AF, atrial fibrillation; CHD, coronary heart disease; HLP, hyperlipidemia; DM, diabetes mellitus; BMI, body mass index; NLR, neutrophil-to-lymphocyte ratio; LMR, lymphocyte-to-monocyte ratio; SII, systemic inflammatory index; PLR, platelet-to-lymphocyte ratio; HALP, hemoglobin, albumin, lymphocyte, platelet score; RBC, red blood cell; HGB, hemoglobin; HCT, hematocrit; MCHC, mean corpuscular hemoglobin concentration; RDW-CV, red blood cell distribution width-coefficient of variation; TC, total cholesterol; TG, triglyceride; LDL-C, low-density lipoprotein cholesterol; HDL-C, high-density lipoprotein cholesterol; non-HDL-C, non-high-density lipoprotein cholesterol; AIP, atherogenic index of plasma; AC, atherogenic coefficient; LCI, lipoprotein combine index; CRI-I, Castelli’s index-I; CRI-II, Castelli’s index-II; FBG, fasting blood glucose; TyG, triglyceride-glucose index; K, potassium; Na, sodium; UA, uric acid; PTA, prothrombin activity; TT, thrombin time; INR, international normalized ratio; APTT, activated partial thromboplastin time; PT, prothrombin time; FIB, fibrinogen; bold font indicates statistically significant differences.

**Table 4:** Baseline characteristics of independent validation dataset.

Variables	Non-comorbidity (N = 57)	Comorbidity (N = 51)	*P*
Age (years)	67 (58, 77)	71 (63, 79)	0.162
Gender (Male, n, %)	36 (63)	31 (61)	0.956
Marriage (Other status, n, %)	3 (5)	4 (8)	0.705
Nationality (Ethnic minority, n, %)	2 (4)	0 (0)	0.497
APT, n (Yes, %)	8 (14)	11 (22)	0.439
Antihypertensive therapy, n (Yes, %)	31 (54)	37 (73)	0.08
Antidiabetic therapy, n (Yes, %)	9 (16)	9 (18)	1
Statins therapy, n (Yes, %)	8 (14)	11 (22)	0.439
Urate-lowering therapy, n (Yes, %)	0 (0)	1 (2)	0.472
Time of onset (h)	48 (24, 168)	48 (19.5, 204)	-
TOAST, n (%)			-
LAA	10 (18)	12 (24)	
SAO	20 (35)	31 (61)	
CE	3 (5)	4 (8)	
SOE	2 (4)	3 (6)	
SUE	1 (2)	1 (2)	
GCS_admission	15 (15, 15)	15 (15, 15)	-
mRS_admission	1 (0,1)	1 (0,2)	-
NIHSS_admission	2 (0, 3)	2 (0, 4)	-
SBP (mmHg)	141 ± 25	141 ± 21	0.995
DBP (mmHg)	87 ± 16	84 ± 13	0.304
Drinking (Yes, n, %)	9 (16)	13 (25)	0.312
Smoking (Yes, n, %)	6 (11)	7 (14)	0.831
HTN (Yes, n, %)	43 (75)	42 (82)	0.522
DM (Yes, n, %)	15 (26)	13 (25)	1
AF (Yes, n, %)	2 (4)	6 (12)	0.145
CHD (Yes, n, %)	3 (5)	6 (12)	0.302
HLP (Yes, n, %)	27 (47)	29 (57)	0.428
BMI (Kg/m^2)	26.18 (24.11, 27.34)	25.80 (23.75, 27.40)	0.667
WBC (10^9/L)	6.7 (5.6, 8.5)	6.7 (5.6, 8.4)	0.735
NEU (10^9/L)	4.39 (3.41, 6.43)	4.42 (3.29, 5.96)	0.453
LYM (10^9/L)	1.46 ± 0.50	1.63 ± 0.60	0.111
MON (10^9/L)	0.65 (0.37, 0.82)	1.01 (0.46, 1.71)	**0.009**
NLR	3.70 (2.29, 4.83)	2.92 (2.00, 3.98)	0.11
LMR	1.96 (1.00, 3.81)	1.00 (1.00, 2.88)	0.057
SII	693 (444, 1048)	558 (381, 749)	0.167
SIRI	2.33 (1.06, 3.94)	2.92 (1.33, 4.93)	0.372
PNR	44.79 (28.18, 59.63)	44.34 (35.01, 56.94)	0.396
PLR	137.14 (104.33, 178.89)	126.34 (99.68, 169.12)	0.304
MHR	0.44 (0.28, 0.71)	0.81 (0.44, 1.22)	**0.002**
NHR	3.50 (2.61, 5.32)	3.77 (2.66, 5.30)	0.758
PHR	152.82 (107.33, 208.59)	180.58 (125.79, 229.65)	0.13
HRR	9.69 ± 1.97	9.95 ± 1.95	0.491
HALP	36.96 (25.31, 52.14)	45.34 (27.60, 57.08)	0.148
RBC (10^12/L)	4.31 (3.76, 4.77)	4.43 (3.92, 4.88)	0.489
HGB (g/L)	130 ± 21	132 ± 21	0.614
HCT (%)	39.14 ± 6.16	39.62 ± 6.23	0.69
MCV (fL)	91.9 ± 5.2	91.8 ± 4.4	0.939
MCHC (g/L)	331 ± 10	333 ± 13	0.519
MCH (pg)	30.5 ± 2.1	30.6 ± 1.7	0.772
RDW-CV (%)	13.3 (12.9, 13.9)	13.2 (12.8, 14.1)	0.5
PLT (10^9/L)	195 (161, 234)	191 (168, 246)	0.59
CRP (mg/L)	4.86 (1.67, 14.24)	3.14 (0.94, 14.24)	0.488
TC (mmol/L)	4.61 (3.89, 5.15)	3.97 (3.04, 5.03)	**0.042**
TG (mmol/L)	1.47 (0.93, 1.83)	1.55 (1.02, 2.55)	0.35
LDL-C (mmol/L)	2.81 ± 0.97	2.44 ± 0.89	**0.041**
HDL-C (mmol/L)	1.28 (1.12, 1.50)	1.21 (0.98, 1.42)	0.102
Non-HDL-C (mmol/L)	3.08 (2.66, 3.80)	2.68 (2.05, 3.61)	0.072
AIP	0.03 ± 0.27	0.14 ± 0.28	0.048
AC	2.50 (1.82, 3.08)	2.32 (1.79, 3.07)	0.629
LCI	12.24 (7.59, 22.75)	11.65 (6.01, 21.04)	0.582
CRI-I	3.50 (2.82, 4.08)	3.32 (2.80, 4.07)	0.629
CRI-II	2.17 ± 0.72	2.07 ± 0.69	0.439
FBG (mmol/L)	6.27 (5.46, 7.71)	5.94 (5.11, 7.52)	0.447
TyG	8.94 (8.56, 9.28)	8.93 (8.48, 9.38)	0.588
UREA (mmol/L)	6.26 (4.71, 7.19)	6.60 (5.28, 8.37)	**0.045**
CREA (μmol/L)	64.0 (54.8, 72.8)	85.9 (71.3,100.0)	**< 0.001**
UA_admission (μmol/L)	311.3 ± 106.0	463.9 ± 66.7	**< 0.001**
UA_3d (μmol/L)	264 (197, 361)	478 (432, 523)	**< 0.001**
K (mmol/L)	3.86 ± 0.38	3.84 ± 0.44	0.787
Na (mmol/L)	140.8 (139.0, 142.0)	140.7 (139.5, 142.5)	0.653
Cl (mmol/L)	105.7 (104.2, 107.5)	105.3 (102.6, 107.1)	0.21
PTA (%)	111 ± 18	109 ± 22	0.577
TT (s)	17.0 ± 1.6	17.2 ± 1.4	0.507
INR	0.98 (0.93, 1.03)	0.98 (0.94, 1.03)	0.88
APTT (s)	28.7 (26.9, 30.8)	28.6 (26.7, 30.7)	1
PT (s)	11.1 (10.4, 12.0)	11.1 (10.6, 11.8)	0.56
FIB (g/L)	3.27 (2.70, 4.38)	3.12 (2.63, 3.88)	0.473

APT, antiplatelet therapy; SBP, systolic blood pressure; DBP, diastolic blood pressure; LAA, large-artery atherosclerosis; SAO, small artery occlusion; CE, Cardioembolism; SOE, stroke of other determined etiologies; SUE, stroke of undetermined etiologies; GCS, Glasgow Coma Scale; mRS, Modified Rankin Scale; NIHSS, National Institutes of Health Stroke Scale; HTN, hypertension; AF, atrial fibrillation; CHD, coronary heart disease; HLP, hyperlipidemia; DM, diabetes mellitus; BMI, body mass index; NLR, neutrophil-to-lymphocyte ratio; LMR, lymphocyte-to-monocyte ratio; SII, systemic inflammatory index; PLR, platelet-to-lymphocyte ratio; HALP, hemoglobin, albumin, lymphocyte, platelet score; RBC, red blood cell; HGB, hemoglobin; HCT, hematocrit; MCHC, mean corpuscular hemoglobin concentration; RDW-CV, red blood cell distribution width-coefficient of variation; TC, total cholesterol; TG, triglyceride; LDL-C, low-density lipoprotein cholesterol; HDL-C, high-density lipoprotein cholesterol; non-HDL-C, non-high-density lipoprotein cholesterol; AIP, atherogenic index of plasma; AC, atherogenic coefficient; LCI, lipoprotein combine index; CRI-I, Castelli’s index-I; CRI-II, Castelli’s index-II; FBG, fasting blood glucose; TyG, triglyceride-glucose index; K, potassium; Na, sodium; UA, uric acid; PTA, prothrombin activity; TT, thrombin time; INR, international normalized ratio; APTT, activated partial thromboplastin time; PT, prothrombin time; FIB, fibrinogen. Bold font indicates statistically significant differences.

### Univariate and multivariate analyses for HUA-IS comorbidity

**Figure 2:**
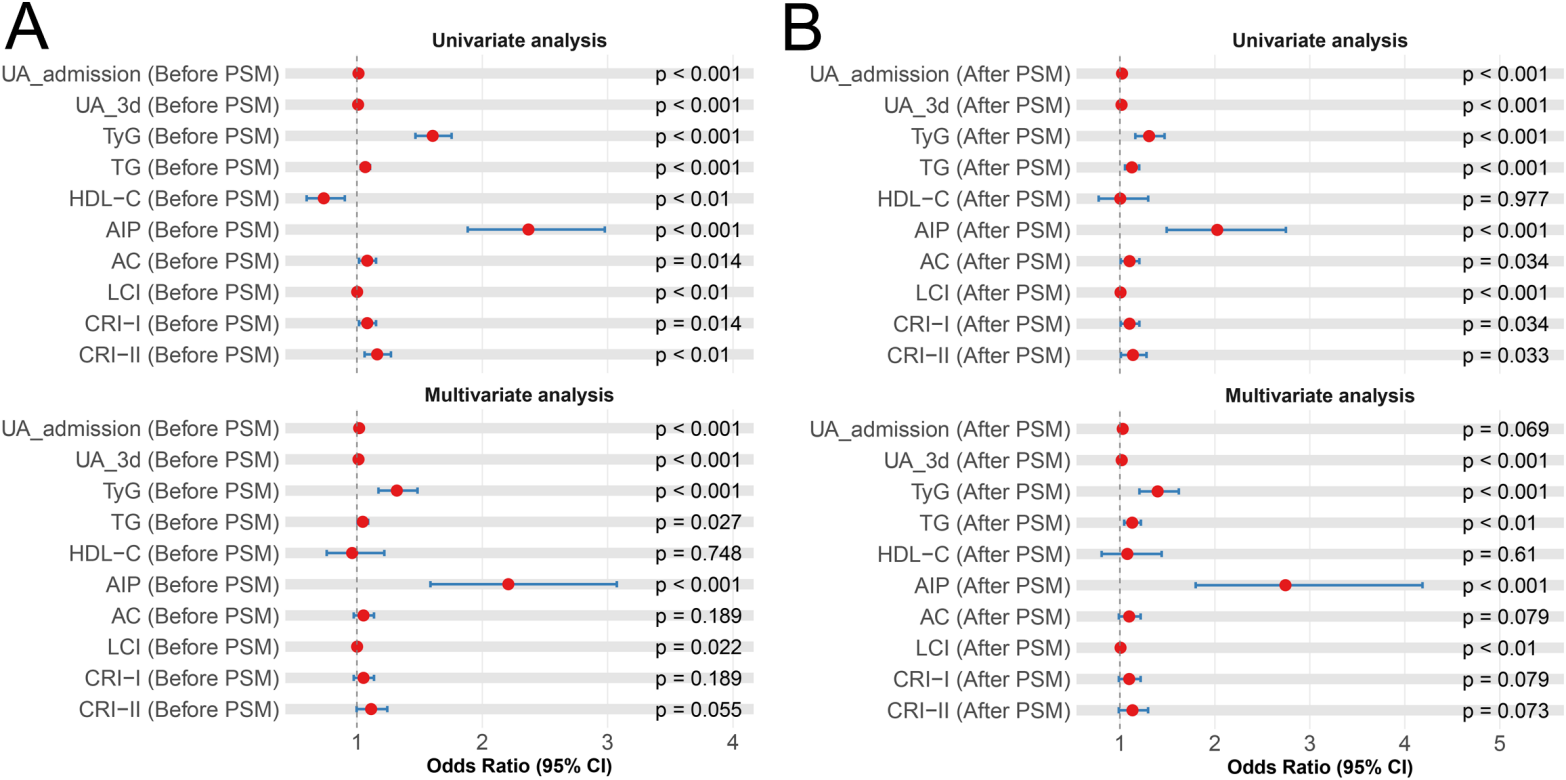
Forest plot displays the results of univariate and multivariate analyses assessing the associations between metabolic parameters and the risk of HUA-IS comorbidity **(A)** pre-PSM and **(B)** post-PSM. IS, ischemic stroke; HUA, hyperuricemia; OR, odd ratio; PSM, propensity score matching.

Ten metabolic indicators (lipid, glucose, and uric acid metabolism) were included, followed by univariate and multivariate analyses. Before PSM, UA_admission (OR: 1.02, 95% CI: 1.02-1.02, *P* < 0.001), UA_3d (OR: 1.01, 95% CI: 1.01-1.01, *P* < 0.001), TyG (OR: 1.32, 95% CI: 1.17-1.48, *P* < 0.001), TG (OR: 1.05, 95% CI: 1.01-1.09, *P* = 0.027), AIP (OR: 2.21, 95% CI: 1.59-3.07, *P* < 0.001), and LCI (OR: 1.00, 95% CI: 1.00-1.00, *P* = 0.022) showed significant associations with the comorbidity in multivariate models. After PSM, only five metabolic parameters, namely, UA_3d (OR: 1.02, 95% CI: 1.02-1.02, *P* < 0.001), TyG (OR: 1.40, 95% CI: 1.21-1.62, *P* < 0.001), TG (OR: 1.13, 95% CI: 1.05-1.22, *P* = 0.002), AIP (OR: 2.74, 95% CI: 1.80-4.19, *P* < 0.001), and LCI (OR: 1.01, 95% CI: 1.00-1.01, *P* = 0.001) were significantly associated with increased risk of HUA-IS comorbidity in fully adjusted models. All associations were statistically significant (*P* < 0.05), visually summarized in **Figures 2A, B**, **Supplementary Table S7**.

### Association of lipid parameters with HUA-IS comorbidity

**Figure 3:**
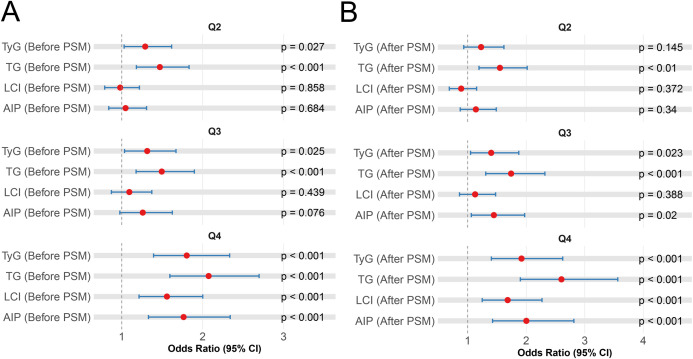
Assessment of the associations between metabolic parameter quartiles and the risk of HUA-IS comorbidity using fully adjusted multivariate models **(A)** pre-PSM and **(B)** post-PSM. IS, ischemic stroke; HUA, hyperuricemia; PSM, propensity score matching.

Subsequently, we performed quantile stratification on the aforementioned five statistically significant metabolic parameters following PSM (**Supplementary Table S8**). After PSM, the quartiles of UA_3d were not significantly associated with the comorbidity (all *P*-values > 0.05, in all models). Elevated risks of HUA-IS comorbidity were observed in the highest quartile (Q4) of the following indicators in fully adjusted multivariate models: TyG (OR: 1.92, 95% CI: 1.40-2.62, *P* < 0.001), TG (OR: 2.60, 95% CI: 1.90-3.56, *P* < 0.001), AIP (OR: 2.00, 95% CI: 1.42-2.81, *P* < 0.001), LCI(OR: 1.68, 95% CI: 1.25-2.27, *P* = 0.001). Furthermore, significant dose-response associations were confirmed for all above indicators, with a trend *P*-value < 0.05 across increasing quartiles (**Figures 3A, B**, **Supplementary Table S9**).

### RCS analysis

**Figure 4:**
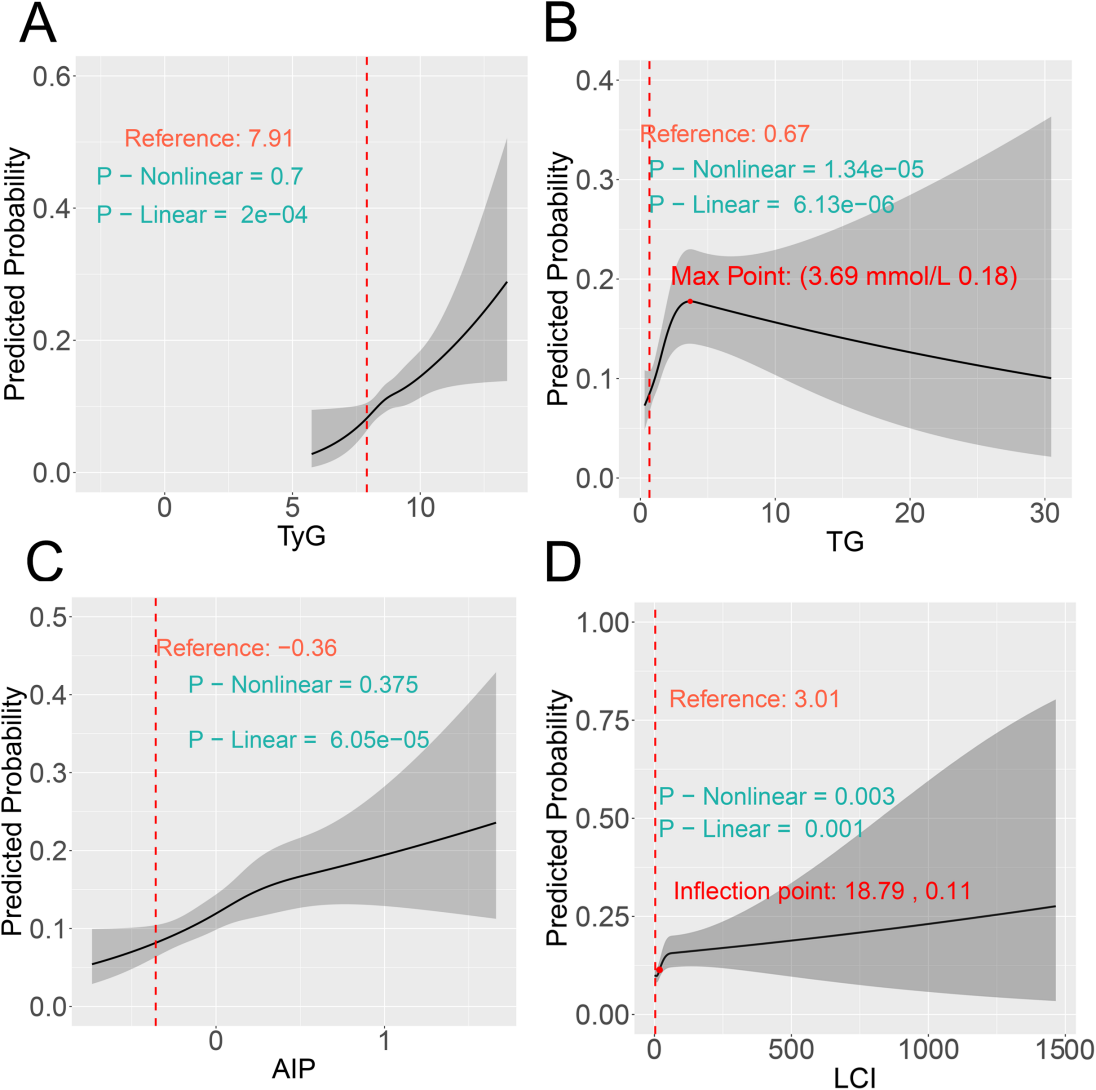
RCS reveals the non-linear associations between metabolic parameters **(A)** TyG, **(B)** TG, **(C)** AIP, and **(D)** LCI and the risk of comorbidity before PSM. RCS, restricted cubic spline; PSM, propensity score matching.

**Figure 5:**
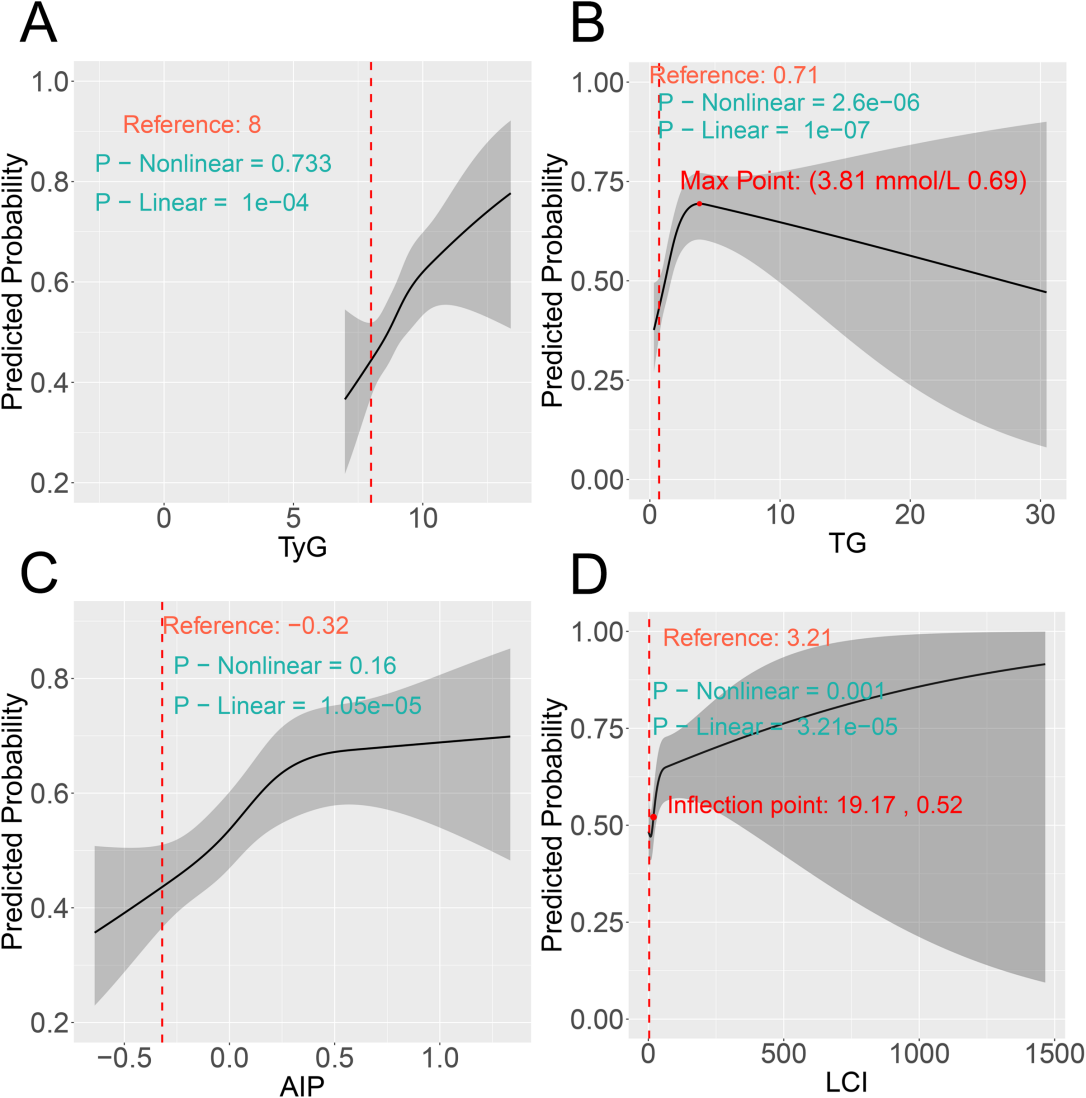
RCS reveals the non-linear associations between metabolic parameters **(A)** TyG, **(B)** TG, **(C)** AIP, and **(D)** LCI and the risk of comorbidity after PSM. RCS, restricted cubic spline; PSM, propensity score matching.

Although significant linear associations were observed for four indicators before (**Figure 4**) and after PSM (**Figure 5**), only TG and LCI demonstrated significant nonlinear relationships (nonlinear *P* < 0.05). Specifically, for TG, the risk of comorbidity showed a pronounced increase with increasing TG levels below the threshold of 3.69 mmol/L (pre-PSM) and 3.81 mmol/L (post-PSM). Above these cutoffs (TG > 3.69 and 3.81 mmol/L), the risk continued to decrease (**Figure 4B**, **Figure 5B**). To verify whether the inverted U-shaped effect was due to the small TG sample, we identified subsets with TG ≥ 3.69 (pre-PSM) and ≥ 3.81 (post-PSM) mmol/L, which included 360 patients (8%) and 160 patients (8%), respectively. Excluding these patients, repeated RCS analyses showed the nonlinear relationship was no longer significant (nonlinear *P*-values: 0.544 and 0.246, **Supplementary Figures S3A**, **B**). Thus, the U-shaped effect may reflect the sparse high-TG sample rather than a true biological effect. Besides, regarding the LCI, its inflection points were 18.79 (before PSM) and 19.17 (following PSM), respectively. When LCI values were below these thresholds, the risk of comorbidity increased in a statistically significant manner with the elevation of LCI; once LCI exceeded these inflection points, the rate of risk elevation began to decelerate, reflecting a nonlinear dose-response relationship between LCI and comorbidity risk (**Figures 4D**, **5D**).

### Clinlabomics models of comorbidity

**Figure 6:**
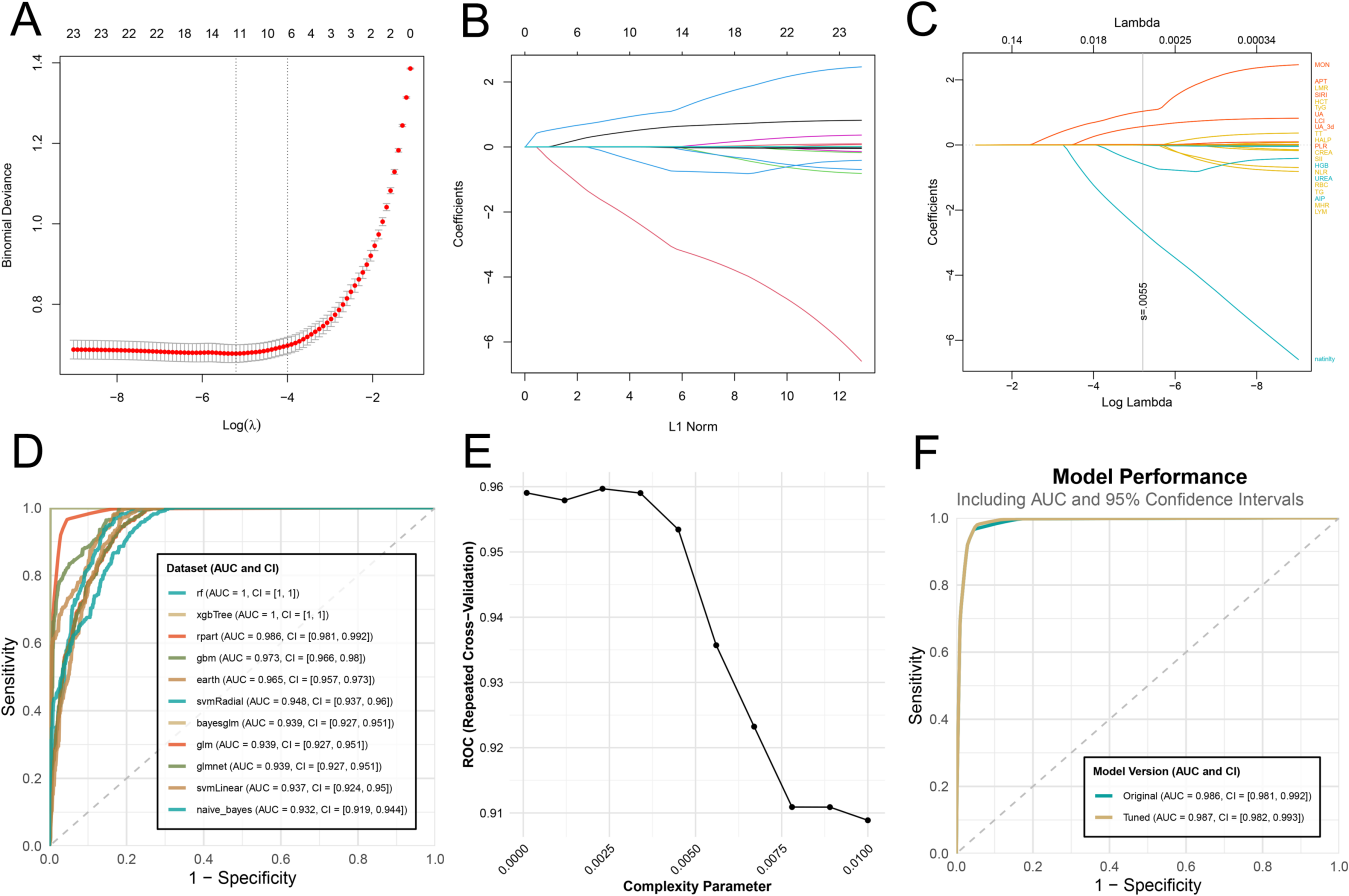
Feature selection, Clinlabomics model development, and optimal Clinlabomics model. **(A)** Regularization parameter selection for LASSO regression. The X-axis represents log(λ), where λ refers to the regularization parameter of LASSO. The Y-axis represents Binomial Deviance, a metric for assessing model fitting performance, smaller values indicate better concordance between the model and the data. The numbers above the plot represent the count of feature variables retained by the model at the corresponding log(λ) values. **(B)** Variable coefficient trajectories derived from LASSO regression, which illustrates the dynamic changes in the regression coefficients of each feature as the regularization intensity varies. The X-axis represents L1 Norm, corresponding to the intensity of the LASSO regularization term; a larger L1 norm indicates stronger regularization constraints, leading to greater shrinkage of feature coefficients. The Y-axis represents Coefficients, representing the coefficient values of individual features in the model. **(C)** Variables with positive, zero, and negative coefficients identified via LASSO analysis are labeled with orange, yellow, and blue, respectively, with specific variables displayed on the right. The gray line (s=0.0055) indicates a critical value of the penalty parameter *λ*, marking the threshold for optimal variable selection. **(D)** ROC curves of Clinlabomics models developed by 11 different ML algorithms. **(E)** Visualization of the hyperparameter tuning process and determination of optimal hyperparameters for the Clinlabomics model constructed using the optimal rpart algorithm. **(F)** ROC curves of the optimal rpart algorithm with and without hyperparameter tuning. LASSO, Least Absolute Shrinkage and Selection Operator; ROC, receiver operating characteristic; ML, machine learning.

**Figure 7:**
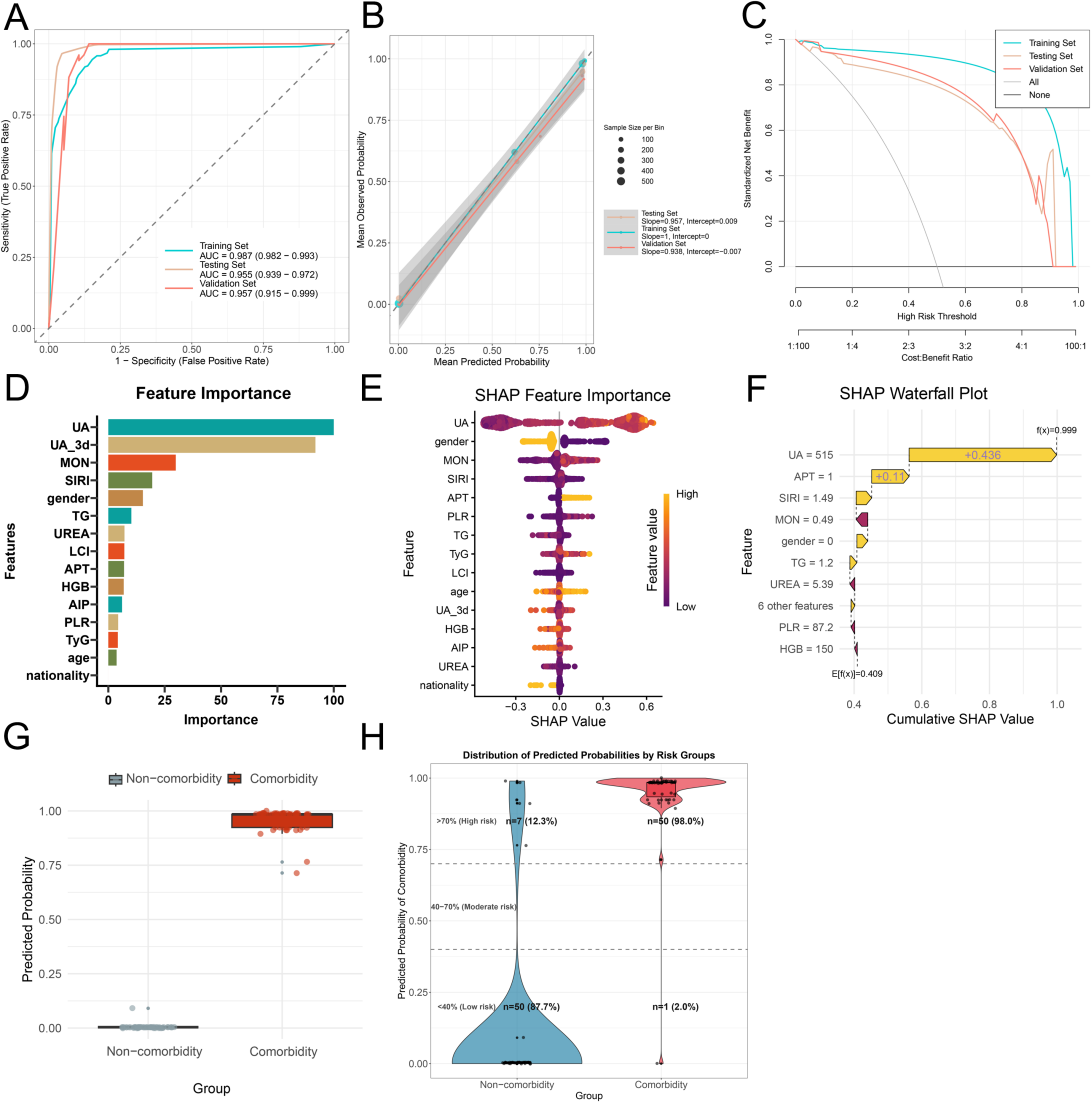
Optimal Clinlabomics model, its interpretation, and discriminative ability. **(A)** ROC curves and corresponding AUC values, **(B)** Calibration curves, and **(C)** DCA curves of the Clinlabomics model (built with optimal hyperparameters) across three datasets. **(D)** Feature importance ranking of the included variables. **(E)** SHAP interpretation of the included variables, which illustrates the magnitude, direction, and association between feature values and their contributions to the model. The y-axis lists features ranked by their impact; the x-axis (SHAP value) reflects the direction and strength of a feature’s contribution to the model’s estimate (positive SHAP values increase comorbidity risk, while negative values decrease it); colors denote the feature’s own value level (orange = high feature value, purple = low feature value), and each dot represents the contribution of the feature in a single sample. **(F)** SHAP waterfall plot illustrates the step-by-step contribution of each feature to the model’s estimate for a single sample. Each feature’s block (yellow = positive contribution, purple = negative contribution) corresponds to its SHAP value, representing how much the feature shifts the estimate away from the average level. Ultimately, the total positive contributions outweigh the negative ones, resulting in the sample’s estimated probability (0.999) being substantially higher than the model’s mean estimate. **(G)** Probability distribution of the validation set (labels removed) via the optimal model. **(H)** The probability distribution of the optimal model accurately identifies non-comorbidity (probability < 40%) and comorbidity (probability > 70%) groups in the independent validation dataset. UA, represents UA_admission.

A total of 23 differentially expressed variables from the training set were subjected to LASSO regression analysis, which identified 11 feature variables for subsequent model development (**Figures 6A-C**). However, considering the associations of age, gender, TyG, and TG with the comorbidity, we incorporated these variables into subsequent model construction. Eleven​ algorithms were used to develop Clinlabomics models based on the training set, with the optimal Clinlabomics model (established by the rpart algorithm) identified (F1-score: 0.960; sensitivity: 0.965; specificity: 0.954; AUC: 0.986, 95% CI: 0.981-0.992) (**Figure 6D**). Subsequently, in the training set, hyperparameter tuning was conducted on the optimal model, with the complete tuning procedure depicted in **Figure 6E**. The optimal hyperparameter for the rpart algorithm was identified as cp=0.0017. In the training set, ROC curves were generated and AUC values were calculated for the untuned and tuned models, demonstrating a slightly higher AUC in the tuned model (0.987, 95% CI: 0.982-0.993) compared to the untuned model (0.986, 95% CI: 0.981-0.992) (**Figure 6F**). Ultimately, the optimally tuned Clinlabomics model was selected, with ROC curves generated across the training set, testing set (AUC: 0.955, 95% CI: 0.939-0.972), and validation set (AUC: 0.957, 95% CI: 0.915-0.999) (**Figure 7A**). Calibration curves demonstrated that, for the training, testing, and validation sets, the slopes of the relationship between estimated and observed probabilities approximated to 1 (with corresponding intercepts near 0), indicating robust calibration performance across these datasets and high concordance between estimated and actual event probabilities (**Figure 7B**). In addition, the Brier score was low for the training (0.034), testing (0.084), and validation (0.068) sets, indicating the model’s accurate assessment with minimal bias across these datasets. Furthermore, for the three datasets, their DCA curves exhibited significantly higher standardized net benefit than the “none” (no risk stratification) strategy across a wide high-risk threshold range, indicating the model’s robust clinical decision-making net benefit across these datasets (**Figure 7C**). Subsequently, feature importance ranking (**Figure 7D**) and SHAP-based interpretation (**Figure 7E**) were performed for the optimal model, revealing the UA_admission, UA_3d, TG, LCI, AIP, TyG as the influential metabolic factors. Randomized​ SHAP visualization of a subject mitigated the model’s black-box limitation by elucidating impactful variables, aiding individualized diagnosis and treatment (**Figure 7F**). Subsequently, the validation set was used as a new dataset. Without group labels, it was assessed by the model, classifying 57 as comorbidity and 51 as non-comorbidity (**Figure 7G**). Probabilities < 40%, 40–70%, and >70% were defined as low-risk, intermediate-risk, and high-risk, respectively. Comparison with original labels showed that 50 (87.7%, probability < 40%) non-comorbidity and 50 (98.0%, probability > 70%) comorbidity patients were accurately identified (**Figure 7H**), indicating favorable discriminative ability of our optimal Clinlabomics model. To identify comorbid populations from healthy controls, we excluded hyperuricemia-negative IS patients from the non-comorbid group and analyzed the data via the aforementioned methods. The rpart algorithm remained optimal (**Supplementary Figure 4A**), with an optimal cp of 0.00015 after tuning (**Supplementary Figure 4B**), though the adjusted model’s AUC did not differ from the unadjusted model’s AUC (**Supplementary Figure 4 C**). The optimal Clinlabomics model exhibited AUCs of 0.974 (95%CI: 0.968–0.980), 0.916 (95%CI: 0.896–0.937), and 0.976 (95%CI: 0.949–1.00) across three datasets, with corresponding Brier scores of 0.051, 0.093, and 0.087 (**Supplementary Figure 5A**). Calibration and DCA curves (**Supplementary Figures 5B, C**) confirmed its strong performance in distinguishing comorbid patients from healthy controls. Feature importance ranking and SHAP plots highlighted UA, UA_3d, LCI, AIP, TyG, and TG as key predictors (**Supplementary Figures 5D-F**). In the validation set (72 unlabeled subjects), the model identified 45 comorbid patients and 27 healthy controls (**Supplementary Figure 5G**), with correct classification rates of 95.2% (20/21) for healthy controls and 78.4% (40/51) for comorbid patients (**Supplementary Figure 5H**). Meanwhile, we also attempted to build models for the IS population, but failed due to overfitting of ML algorithms, so no IS-specific comorbidity model was established.

## Discussion

This study identified critical metabolic indicators (UA_3d, TyG, TG, AIP, and LCI), associated with HUA-IS comorbidity. It further constructed Clinlabomics models employing 11 advanced machine learning algorithms and ascertained the optimal model. Furthermore, through feature importance analysis and SHAP interpretation, we addressed the inherent “black-box” constraints of machine learning-driven modeling. Leveraging these integrated analyses, we identified metabolic parameters strongly linked to HUA-IS comorbidity, specifically TyG, TG, AIP, and LCI.

Hyperuricemia (HUA), a prevalent metabolic abnormality, is of significant value​ in patients with IS. Şengüldür et al. revealed that HUA increases the risk of IS by 2.4-fold (OR: 2.402, 95% CI: 1.792–3.221) (43). Besides, HUA is also linked to delayed neurological recovery, elevated risk of recurrent stroke, and increased mortality (44), making it a clinically important issue in cerebrovascular diseases (CVD). In terms of mortality prediction, SUA level serves as an independent predictor of in-hospital death in IS patients (hazard ratio [HR]:1.02, 95% CI: 1.00 - 1.03) (45). The risk is markedly heightened when HUA coincides with a high-inflammatory state, resulting in a 4.09-fold increase in mortality and a 98% higher risk of major disability among AIS patients (46). Furthermore, elevated SUA was independently associated with spontaneous hemorrhagic transformation in male IS patients (47). Notably, Mendelian randomization analysis also confirmed that higher SUA levels are positively associated with an increased risk of IS and worse clinical outcomes (48). Interestingly, anti-hyperuricemia treatment offers a potential avenue for improving prognosis. In patients with HTN and HUA, such treatment correlates with a reduced incidence of composite CVD (OR: 0.93, 95% CI: 0.88 - 0.99) (49). Therefore, these findings underscore the critical role of HUA in stroke progression and highlight the potential benefits of urate-lowering therapy in high-risk populations.

Furthermore, the TyG index, derived from FBG and TG, acts as a well-validated surrogate marker for insulin resistance (IR). IR, regarded as one of the core underlying mechanisms of HUA, exacerbates SUA accumulation by impairing renal UA excretion (50). For instance, observational studies have confirmed this positive correlation (23, 51), and findings from the China Health and Retirement Longitudinal Study (CHARLS) further corroborate that an elevated baseline TyG index is significantly linked to a higher incidence risk of HUA (52). Beyond its association with HUA, the TyG index also exhibits a robust relationship with IS risk. Data from the Kailuan Prospective Cohort Study showed that a higher TyG index was associated with a significantly elevated incidence of IS (HR: 1.35, 95% CI: 1.31–1.40) (53). Notably, a meta-analysis of cerebrovascular disease studies further strengthened this link, reporting that a higher TyG index correlated with an increased risk of IS (OR: 1.37, 95% CI: 1.22–1.54) (16). Given its dual associations with HUA and IS, the TyG index may serve as a crucial risk factor for HUA-IS comorbidity, offering a simple and accessible marker to identify individuals at heightened risk of concurrent HUA and IS.

More importantly, HUA also exerts a synergistic effect with dyslipidemia and other metabolic disturbances to drive vascular pathological remodeling, ultimately worsening prognosis in patients with IS. A community-based cross-sectional study conducted among middle-aged and elderly Chinese individuals indicated that non-HDL-C and other conventional lipid profiles, especially TG, are significantly correlated with HUA, suggesting a shared metabolic pathway between lipid dysregulation and SUA elevation (54). Similarly, data from the Korea National Health and Nutrition Examination Survey (KNHANES, 2016–2017) reinforced the association between dyslipidemia, including TG, one of its abnormal components, and elevated SUA levels in adults, further supporting the intrinsic link between lipid parameters and urate homeostasis (55). For instance, a cross-sectional study indicated a positive correlation between AIP and the prevalence of HUA among middle-aged and elderly Chinese individuals, supporting its utility as a discriminative marker (56). Consistent with these findings, Huang et al. validated the estimated value of AIP for HUA using National Health and Nutrition Examination Survey (NHANES) data, reporting a fully adjusted OR of 3.04 (95% CI: 1.93–4.79) (57). Beyond its role in HUA, dyslipidemia is a well-established modifiable risk factor for CVD, including stroke (58). We previously applied an unsupervised clustering algorithm to identify a distinct phenotype of AIS, in which both traditional (TC, TG, HDL-C, and LDL-C) and non-traditional (e.g., AIP, LCI, non-HDL-C, AC, CRI-I, and CRI-II) lipid parameters were significantly elevated (17). Of note, Jia et al. reported that after multivariable adjustment, TG were significantly linked to an increased risk of IS (HR: 1.12 (Q4 vs. Q1), 95%CI: 1.03–1.23; *P*-trend = 0.0095) (59). In recent years, non-traditional lipid profiles primarily serve as independent predictors for CVD (60, 61). Based on a meta-analysis,​​ Liu et al. reported that elevated non-traditional lipid levels are significantly associated with an increased risk of IS (62). Furthermore, non-traditional lipid parameters may predict carotid plaque vulnerability in AIS patients (63). Evidence shows that elevated AIP significantly increases the risk of IS, as demonstrated in long-term retrospective cohorts (64, 65). Furthermore, higher AIP levels are correlated with an increased risk of the large artery atherosclerosis (LAA) subtype (66) and serve as an independent predictor of poor functional outcomes in AIS patients (OR = 1.84, 95% CI: 1.23 - 2.53) (67). In addition, a hospital-based observational study in China indicates that AC and LCI serve as significant predictors of both intracranial and extracranial atherosclerotic stenosis in IS patients (38).

Notably, we used the “Icare” package to build Clinlabomics models using 11 ML algorithms, based on clinical and laboratory indicators, enabling in-depth mining of hidden data insights. Feature importance ranking and SHAP interpretation addressed the traditional “black-box” issue of models established by ML algorithms. Additionally, individual patient SHAP interpretation facilitates personalized and precision medicine. We revealed that the Clinlabomics model developed by the rpart algorithm exhibited the optimal performance. Similarly, Liu et al. demonstrated that the model developed using the rpart algorithm exhibited high accuracy (0.911), sensitivity (0.851), and specificity (0.821) in diagnosing obstructive coronary artery disease (CAD) (68). Furthermore, Shah et al. employed a classification tree model based on the rpart algorithm to identify ocular baseline factors predictive of treatment frequency in patients with neovascular age-related macular degeneration (nAMD) (69). These findings collectively demonstrate that the model developed by the rpart algorithm exhibits favorable discriminative performance. Nevertheless, large-scale​ and prospective multicenter studies are required to validate the generalizability and robustness of this optimal Clinlabomics model.

Notably, admission UA levels, which reflect acute-phase status, are prone to interference from stress, failing to reflect chronic baseline metabolic status, whereas comorbidity risk hinges on long-term metabolic abnormalities. For one thing, insufficient statistical power for admission UA levels arose from incomplete adjustment for potential confounders (e.g. dietary data, genetic susceptibility, etc.). For another, acute-phase UA may merely serve as a marker of IS, reflecting oxidative stress-induced damage (70), rather than a direct pathogenic factor for comorbidities, resulting in no significant association between acute-phase UA and comorbidity. Regarding UA on the third day of hospitalization, its quartile grouping may have failed to capture UA’s threshold effect. However, this negative finding does not negate UA’s potential link to HUA-IS comorbidity but highlights the limited clinical value of acute-phase levels. Future studies should focus on chronic baseline UA (pre-admission measurements) and dynamic UA changes (admission-to-discharge rate of change), alongside more precise confounder adjustment, more sensitive statistical models, multicenter designs with an expanded sample size, and optimized strategies to capture UA’s threshold effect (e.g., refined quartile stratification), to clarify the true association between chronic UA level and comorbidity.

For the RCS curve of TG and HUA-IS comorbidity risk, an inverted U-shaped association was observed. However, after excluding the small sample of patients with TG levels above the threshold, the nonlinear association was no longer statistically significant. These findings suggest that the observed inverted​ U-shaped relationship is primarily attributable to sparse sampling of the high-TG population rather than a true biological effect. Future studies should expand the sample size in prospective studies​ within the high-TG interval (particularly focusing on the hyperlipidemia population) to more reliably validate the validity of this potential association.

However, this study has several limitations. First, despite rigorous adjustment for known confounders, residual confounding may persist due to unmeasured cardiovascular or metabolic risk factors, both of which may influence outcomes through complex biological pathways. Second, as a single-center retrospective study, inherent constraints of this design should be acknowledged: specifically, the selection of controls (stroke-like symptoms but negative imaging findings) may affect the association between metabolic parameters and comorbidities, and while assay standardization was maintained within our center, subtle variability in detection techniques cannot be completely ruled out. Additionally, this retrospective design only captures cross-sectional associations at a specific time point, failing to establish a causal relationship or clarify the temporal sequence between variables. Third, the single-center design limits the generalizability of our findings. Specifically, in multi-center settings, differences across institutions in case mix (e.g., baseline patient characteristics) and standardization of assays may hinder external validity. Last but not least, future studies should address these limitations by adopting large-scale, multi-center prospective cohorts, optimizing control group selection, particularly including asymptomatic healthy controls, to mitigate selection bias, standardizing assay methods across centers, and supplementing key covariates (including comprehensive dietary assessments and UA metabolism-related gene polymorphisms). Such investigations will better elucidate the associations between metabolic parameters and HUA-IS comorbidities, further validate the efficacy of these parameters for risk assessment, and thereby generate more robust clinical evidence to guide clinical practice.

In real-world clinical practice, the Clinlabomics model constructed based on metabolic and routine clinical indicators, enables rapid screening for HUA-IS comorbidity in suspected patients during early hospitalization, effectively addressing the issue of missed diagnoses in primary hospitals. Second, individualized intervention can be achieved through risk stratification: high-risk patients are prioritized to receive targeted therapy, while low-risk patients undergo routine follow-up, thereby enhancing the efficiency of clinical management. Finally, these metabolic indicators can serve as markers for monitoring treatment efficacy, thereby establishing a closed-loop management framework of “screening-intervention-monitoring.

## Conclusion

In conclusion, this study identified TyG, TG, AIP, and LCI as metabolic parameters significantly associated with HUA-IS comorbidity, developed an optimal Clinlabomics model based on the rpart algorithm for risk assessment, and revealed their critical role in comorbidity. This provided new insights for comorbidity research regarding metabolic biomarkers. However, further validation through large-scale, multi-center prospective cohorts is essential to confirm the generalizability and their clinical utility for risk assessment and treatment guidance for HUA-IS comorbidity.

## Data Availability

The original contributions presented in the study are included in the article/**Supplementary Material**. Further inquiries can be directed to the corresponding author.
