# Supplementary material for: Identifying metabolic parameters as key indicators of hyperuricemia and ischemic stroke comorbidity via interpretable Clinlabomics models
Source: Front Endocrinol (Lausanne). 2026 Jan 13;16:1737419. doi: 10.3389/fendo.2025.1737419 (PMC12834788; doi:10.3389/fendo.2025.1737419)
Supplement: Supplementary file 1 [file Table1.docx]

**Table S1 Missing proportions of variables, and their distribution before and imputation.**

| Variables | Missing value (%) | Eliminating NA | Imputation NA | P-value |
| --- | --- | --- | --- | --- |
| BMI (Kg/m^2) | 208 (4.4) | 25.12 (23.02, 27.1) | 25.14 (23.01, 27.1) | 0.931 |
| WBC (10^9/L) | 46 (0.9) | 6.7 (5.5, 8.2) | 6.7 (5.5, 8.2) | 0.942 |
| NEU (10^9/L) | 46 (0.9) | 4.40 (3.34, 5.93) | 4.41 (3.34, 5.94) | 0.909 |
| LYM (10^9/L) | 46 (0.9) | 1.46 (1.08, 1.86) | 1.46 (1.08, 1.86) | 0.915 |
| MON (10^9/L) | 46 (0.9) | 0.51 (0.38, 0.83) | 0.51 (0.38, 0.83) | 0.986 |
| RBC (10^12/L) | 46 (0.9) | 4.33 (3.9, 4.77) | 4.33 (3.9, 4.77) | 0.866 |
| HGB (g/L) | 46 (0.9) | 132 (118, 144) | 132 (118, 144) | 0.939 |
| HCT (%) | 46 (0.9) | 39.7 (35.9, 43.5) | 39.7 (35.9, 43.5) | 0.95 |
| MCV (fL) | 46 (0.9) | 92.4 (89.2, 95.5) | 92.4 (89.2, 95.5) | 0.989 |
| MCHC (g/L) | 46 (0.9) | 331 (324, 337) | 331 (324, 337) | 0.916 |
| MCH (pg) | 46 (0.9) | 30.6 (29.4, 31.8) | 30.6 (29.4, 31.8) | 1 |
| RDW-CV (%) | 46 (0.9) | 13.3 (12.8, 14.1) | 13.3 (12.8, 14.1) | 0.972 |
| PLT (10^9/L) | 46 (0.9) | 186 (146, 227) | 186 (146, 226.5) | 0.992 |
| CRP (mg/L) | 185 (4) | 3.7 (1.02, 6.45) | 3.7 (1.03, 6.46) | 0.828 |
| TC (mmol/L) | 9 (0.1) | 4.46 (3.71, 5.24) | 4.46 (3.71, 5.24) | 0.968 |
| TG (mmol/L) | 9 (0.1) | 1.45 (1.00, 2.13) | 1.45 (1.00, 2.13) | 0.973 |
| LDL-C (mmol/L) | 9 (0.1) | 2.68 (2.15, 3.27) | 2.68 (2.15, 3.27) | 0.977 |
| HDL-C (mmol/L) | 9 (0.1) | 1.25 (1.05, 1.48) | 1.25 (1.05, 1.48) | 0.972 |
| FBG (mmol/L) | 32 (0.6) | 5.83 (5.01, 7.52) | 5.84 (5.01, 7.52) | 0.937 |
| K (mmol/L) | 55 (1.1) | 3.86 (3.62, 4.12) | 3.86 (3.62, 4.12) | 0.992 |
| Na (mmol/L) | 55 (1.1) | 140.6 (138.9, 142.1) | 140.6 (138.9, 142.1) | 0.99 |
| Cl (mmol/L) | 55 (1.1) | 105 (103, 107) | 105 (103, 107) | 0.972 |
| PTA (%) | 185 (4) | 112 (99, 126) | 112 (99, 126) | 0.877 |
| TT (s) | 185 (4) | 16.8 (15.6, 17.8) | 16.8 (15.6, 17.8) | 0.987 |
| INR | 185 (4) | 0.98 (0.93, 1.03) | 0.98 (0.93, 1.03) | 0.881 |
| APTT (s) | 185 (4) | 28.2 (26.4, 30.3) | 28.2 (26.4, 30.3) | 0.955 |
| PT (s) | 185 (4) | 11.0 (10.5, 11.6) | 11.0 (10.5, 11.6) | 0.869 |
| FIB (g/L) | 185 (4) | 2.92 (2.46, 3.59) | 2.92 (2.46, 3.59) | 0.944 |
