# Supplementary material for: Identifying metabolic parameters as key indicators of hyperuricemia and ischemic stroke comorbidity via interpretable Clinlabomics models
Source: Front Endocrinol (Lausanne). 2026 Jan 13;16:1737419. doi: 10.3389/fendo.2025.1737419 (PMC12834788; doi:10.3389/fendo.2025.1737419)
Supplement: Supplementary file 2 [file Table2.docx]

**Table S2 Comparison of baseline characteristics between non-HUA IS and non-HUA HCs.**

| **Variables** | **Non-HUA HCs**  **(n = 1,145)** | **Non-HUA IS**  **(n = 1,082)** | ***P*** |
| --- | --- | --- | --- |
| Age (years) | 64 (56, 72) | 71 (60, 78) | < 0.001 |
| Gender (Male, n, %) | 494 (43) | 675 (62) | < 0.001 |
| Marriage (Other status, n, %) | 86 (8) | 116 (11) | 0.01 |
| Nationality (Ethnic minority, n, %) | 92 (8) | 1 (0) | < 0.001 |
| APT, n (Yes, %) | 10 (1) | 182 (17) | < 0.001 |
| Antihypertensive therapy, n (Yes, %) | 165 (14) | 642 (59) | < 0.001 |
| Antidiabetic therapy, n (Yes, %) | 34 (3) | 241 (22) | < 0.001 |
| Statins therapy, n (Yes, %) | 8 (1) | 182 (17) | < 0.001 |
| Urate-lowering therapy, n (Yes, %) | 0 (0) | 0 (0) | 0.486 |
| SBP (mmHg) | 132 (119, 143) | 141 (128, 157) | < 0.001 |
| DBP (mmHg) | 80 (73, 87) | 83 (75, 94) | < 0.001 |
| Drinking (Yes, n, %) | 98 (9) | 224 (21) | < 0.001 |
| Smoking (Yes, n, %) | 75 (7) | 128 (12) | < 0.001 |
| HTN (Yes, n, %) | 495 (43) | 731 (68) | < 0.001 |
| DM (Yes, n, %) | 141 (12) | 273 (25) | < 0.001 |
| AF (Yes, n, %) | 4 (0) | 40 (4) | < 0.001 |
| CHD (Yes, n, %) | 36 (3) | 69 (6) | < 0.001 |
| HLP (Yes, n, %) | 510 (45) | 572 (53) | < 0.001 |
| BMI (Kg/m^2) | 23.88 (22.22, 26.03) | 25.86 (23.97, 27.30) | < 0.001 |
| WBC (10^9/L) | 6.2 (5.1, 7.6) | 7.0 (5.7, 8.6) | < 0.001 |
| NEU (10^9/L) | 3.98 (2.92, 5.46) | 4.8 (3.65, 6.49) | < 0.001 |
| LYM (10^9/L) | 1.47 (1.08, 1.87) | 1.37 (1.03, 1.72) | < 0.001 |
| MON (10^9/L) | 0.42 (0.34, 0.54) | 0.81 (0.47, 1.41) | < 0.001 |
| NLR | 2.55 (1.74, 4.50) | 3.48 (2.37, 5.46) | < 0.001 |
| LMR | 3.42 (2.36, 4.61) | 1.00 (1.00, 2.89) | < 0.001 |
| SII | 488 (305, 829) | 665 (426, 1059) | < 0.001 |
| SIRI | 1.12 (0.67, 2.19) | 3.58 (1.72, 5.45) | < 0.001 |
| PNR | 47.12 (32.17, 65.43) | 39.22 (27.62, 52.63) | < 0.001 |
| PLR | 125.47 (93.02, 169.47) | 140.40 (105.51, 189.40) | < 0.001 |
| MHR | 0.34 (0.24, 0.46) | 0.64 (0.39, 1.12) | < 0.001 |
| NHR | 3.08 (2.17, 4.34) | 3.91 (2.78, 5.57) | < 0.001 |
| PHR | 138.33 (103.60, 188.19) | 148.6 (111.89, 196.69) | 0.001 |
| HRR | 9.70 (8.48, 10.92) | 9.65 (8.48, 10.68) | 0.13 |
| HALP | 41.09 (28.6, 56.95) | 36.85 (24.65, 51.21) | < 0.001 |
| RBC (10^12/L) | 4.25 (3.80, 4.65) | 4.26 (3.89, 4.68) | 0.079 |
| HGB (g/L) | 128 (115, 141) | 130 (117, 141) | 0.186 |
| HCT (%) | 39.0 (35.3, 42.7) | 39.2 (35.6, 42.6) | 0.7 |
| MCV (fL) | 93.1 (89.9, 96.0) | 92.2 (88.7, 95.3) | < 0.001 |
| MCHC (g/L) | 330 (323, 336) | 331 (324, 338) | < 0.001 |
| MCH (pg) | 30.7 (29.6, 31.9) | 30.6 (29.4, 31.7) | 0.067 |
| RDW-CV (%) | 13.2 (12.6, 13.9) | 13.4 (12.9, 14.2) | < 0.001 |
| PLT (10^9/L) | 183 (145, 226) | 189 (149, 229) | 0.025 |
| CRP (mg/L) | 3.70 (1.11, 6.04) | 3.31 (0.92, 14.24) | < 0.001 |
| TC (mmol/L) | 4.44 (3.80, 5.14) | 4.38 (3.59, 5.32) | 0.63 |
| TG (mmol/L) | 1.24 (0.94, 1.77) | 1.36 (0.96, 1.98) | < 0.001 |
| LDL-C (mmol/L) | 2.63 (2.21, 3.13) | 2.69 (2.03, 3.33) | 0.29 |
| HDL-C (mmol/L) | 1.29 (1.08, 1.54) | 1.25 (1.05, 1.47) | 0.004 |
| Non-HDL-C (mmol/L) | 3.10 (2.53, 3.72) | 3.14 (2.39, 3.98) | 0.467 |
| AIP | 0 (-0.17, 0.17) | 0.05 (-0.14, 0.23) | < 0.001 |
| AC | 2.37 (1.85, 2.98) | 2.52 (1.90, 3.25) | 0.003 |
| LCI | 11.27 (6.90, 19.2) | 12.98 (6.62, 24.05) | 0.001 |
| CRI-I | 3.37 (2.85, 3.98) | 3.52 (2.90, 4.25) | 0.003 |
| CRI-II | 2.03 (1.62, 2.55) | 2.14 (1.67, 2.75) | < 0.001 |
| FBG (mmol/L) | 5.48 (4.89, 6.54) | 6.51 (5.32, 8.56) | < 0.001 |
| TyG | 8.66 (8.26, 9.07) | 8.91 (8.45, 9.41) | < 0.001 |
| UREA (mmol/L) | 5.97 (4.87, 7.15) | 5.81 (4.66, 7.34) | 0.349 |
| CREA (μmol/L) | 62.0 (52.1, 71.5) | 69.0 (58.0, 83.7) | < 0.001 |
| UA_admission (μmol/L) | 278 (232, 321) | 293 (238, 342) | < 0.001 |
| UA_3d (μmol/L) | 248 (195, 306) | 270 (212, 333) | < 0.001 |
| K (mmol/L) | 3.87 (3.66, 4.09) | 3.81 (3.56, 4.05) | < 0.001 |
| Na (mmol/L) | 140.9 (139.2, 142.2) | 140.2 (138.2, 141.9) | < 0.001 |
| Cl (mmol/L) | 105.6 (104, 107.4) | 104.5 (102.3, 106.5) | < 0.001 |
| PTA (%) | 111 (100, 124) | 112 (98, 126) | 0.913 |
| TT (s) | 16.3 (15.1, 17.4) | 17.1 (15.9, 18.2) | < 0.001 |
| INR | 0.98 (0.94, 1.03) | 0.97 (0.92, 1.03) | < 0.001 |
| APTT (s) | 27.9 (26.3, 30.1) | 28.3 (26.7, 30.4) | < 0.001 |
| PT (s) | 11.0 (10.6, 11.5) | 11.1 (10.5, 11.9) | < 0.001 |
| FIB (g/L) | 2.84 (2.41, 3.43) | 3.12 (2.58, 3.82) | < 0.001 |

APT, antiplatelet therapy; SBP, systolic blood pressure; DBP, diastolic blood pressure; TOAST, Trial of Org 10172 in Acute Stroke Treatment; LAA, large-artery atherosclerosis; SAO, small artery occlusion; CE, Cardioembolism; SOE, stroke of other determined etiologies; SUE, stroke of undetermined etiologies; GCS, Glasgow Coma Scale; mRS, Modified Rankin Scale; NIHSS, National Institutes of Health Stroke Scale; HTN, hypertension; AF, atrial fibrillation; CHD, coronary heart disease; HLP, hyperlipidemia; DM, diabetes mellitus; BMI, body mass index; NLR, neutrophil-to-lymphocyte ratio; LMR, lymphocyte-to-monocyte ratio; SII, systemic inflammatory index; PLR, platelet-to-lymphocyte ratio; HALP, hemoglobin, albumin, lymphocyte, platelet score; RBC, red blood cell; HGB, hemoglobin; HCT, hematocrit; MCHC, mean corpuscular hemoglobin concentration; RDW-CV, red blood cell distribution width-coefficient of variation; TC, total cholesterol; TG, triglyceride; LDL-C, low-density lipoprotein cholesterol; HDL-C, high-density lipoprotein cholesterol; non-HDL-C, non-high-density lipoprotein cholesterol; AIP, atherogenic index of plasma; AC, atherogenic coefficient; LCI, lipoprotein combine index; CRI-I, Castelli's index-I; CRI-II, Castelli's index-II; FBG, fasting blood glucose; TyG, triglyceride-glucose index; K, potassium; Na, sodium; UA, uric acid; PTA, prothrombin activity; TT, thrombin time; INR, international normalized ratio; APTT, activated partial thromboplastin time; PT, prothrombin time; FIB, fibrinogen.
