# Supplementary material for: Identifying metabolic parameters as key indicators of hyperuricemia and ischemic stroke comorbidity via interpretable Clinlabomics models
Source: Front Endocrinol (Lausanne). 2026 Jan 13;16:1737419. doi: 10.3389/fendo.2025.1737419 (PMC12834788; doi:10.3389/fendo.2025.1737419)
Supplement: Supplementary file 3 [file Table3.docx]

**Table S3 Comparison basic characteristics between HUA and non-HUA HCs.**

| **Variables** | **Non-HUA HCs**  **(n = 1,145)** | **HUA HCs**  **(n = 1,314)** | ***P*** |
| --- | --- | --- | --- |
| Age (years) | 64 (56, 72) | 61 (49, 72) | **< 0.001** |
| Gender (Male, n, %) | 494 (43) | 810 (62) | **< 0.001** |
| Marriage (Other status, n, %) | 86 (8) | 146 (11) | **0.003** |
| Nationality (Ethnic minority, n, %) | 92 (8) | 76 (6) | **0.033** |
| APT, n (Yes, %) | 10 (1) | 13 (1) | 0.930 |
| Antihypertensive therapy, n (Yes, %) | 165 (14) | 240 (18) | **0.012** |
| Antidiabetic therapy, n (Yes, %) | 34 (3) | 63 (5) | **0.027** |
| Statins therapy, n (Yes, %) | 8 (1) | 13 (1) | 0.574 |
| Urate-lowering therapy, n (Yes, %) | 0 (0) | 25 (2) | **< 0.001** |
| SBP (mmHg) | 132 (119, 143) | 130 (119, 145) | 0.706 |
| DBP (mmHg) | 80 (73, 87) | 82 (72, 90) | **0.015** |
| Drinking (Yes, n, %) | 98 (9) | 178 (14) | **< 0.001** |
| Smoking (Yes, n, %) | 75 (7) | 116 (9) | **0.042** |
| HTN (Yes, n, %) | 495 (43) | 682 (52) | **< 0.001** |
| DM (Yes, n, %) | 141 (12) | 168 (13) | 0.771 |
| AF (Yes, n, %) | 4 (0) | 16 (1) | **0.030** |
| CHD (Yes, n, %) | 36 (3) | 86 (7) | **< 0.001** |
| HLP (Yes, n, %) | 510 (45) | 774 (59) | **< 0.001** |
| BMI (Kg/m^2) | 23.88 (22.22, 26.03) | 24.77 (22.72, 27.28) | **< 0.001** |
| WBC (10^9/L) | 6.2 (5.1, 7.6) | 6.5 (5.5, 7.9) | **< 0.001** |
| NEU (10^9/L) | 3.98 (2.92, 5.46) | 4.08 (3.21, 5.60) | **0.003** |
| LYM (10^9/L) | 1.47 (1.08, 1.87) | 1.55 (1.17, 1.97) | **< 0.001** |
| MON (10^9/L) | 0.42 (0.34, 0.54) | 0.45 (0.36, 0.56) | **0.005** |
| NLR | 2.55 (1.74, 4.50) | 2.63 (1.75, 3.98) | 0.921 |
| LMR | 3.42 (2.36, 4.61) | 3.50 (2.50, 4.57) | 0.420 |
| SII | 488 (305, 829) | 480 (312, 794) | 0.883 |
| SIRI | 1.12 (0.67, 2.19) | 1.20 (0.74, 2.02) | 0.223 |
| PNR | 47.12 (32.17, 65.43) | 44.88 (32.04, 61.78) | **0.011** |
| PLR | 125.47 (93.02, 169.47) | 119.38 (93.12, 153.10) | **0.007** |
| MHR | 0.34 (0.24, 0.46) | 0.36 (0.27, 0.49) | **< 0.001** |
| NHR | 3.08 (2.17, 4.34) | 3.34 (2.51, 4.62) | **< 0.001** |
| PHR | 138.33 (103.6, 188.19) | 147.87 (115.82, 189.74) | **0.002** |
| HRR | 9.70 (8.48, 10.92) | 10.32 (8.96, 11.37) | **< 0.001** |
| HALP | 41.09 (28.6, 56.95) | 46.48 (33.45, 61.67) | **< 0.001** |
| RBC (10^12/L) | 4.25 (3.80, 4.65) | 4.43 (4.00, 4.90) | **< 0.001** |
| HGB (g/L) | 128 (115, 141) | 137 (120, 148) | **< 0.001** |
| HCT (%) | 39.0 (35.3, 42.7) | 41.0 (36.3, 44.8) | **< 0.001** |
| MCV (fL) | 93.1 (89.9, 96.0) | 92.2 (88.9, 95.4) | **< 0.001** |
| MCHC (g/L) | 330 (323, 336) | 331 (324, 337) | **0.003** |
| MCH (pg) | 30.7 (29.6, 31.9) | 30.4 (29.3, 31.7) | **0.008** |
| RDW-CV (%) | 13.2 (12.6, 13.9) | 13.2 (12.6, 13.9) | 0.502 |
| PLT (10^9/L) | 183 (145, 226) | 183 (147, 218) | 0.797 |
| CRP (mg/L) | 3.70 (1.11, 6.04) | 3.70 (1.05, 6.01) | 0.704 |
| TC (mmol/L) | 4.44 (3.80, 5.14) | 4.52 (3.79, 5.21) | 0.068 |
| TG (mmol/L) | 1.24 (0.94, 1.77) | 1.56 (1.08, 2.36) | **< 0.001** |
| LDL-C (mmol/L) | 2.63 (2.21, 3.13) | 2.72 (2.19, 3.31) | 0.089 |
| HDL-C (mmol/L) | 1.29 (1.08, 1.54) | 1.22 (1.05, 1.46) | **< 0.001** |
| Non-HDL-C (mmol/L) | 3.10 (2.53, 3.72) | 3.26 (2.55, 3.91) | **< 0.001** |
| AIP | 0 (-0.17, 0.17) | 0.1 (-0.06, 0.3) | **< 0.001** |
| AC | 2.37 (1.85, 2.98) | 2.66 (2.03, 3.28) | **< 0.001** |
| LCI | 11.27 (6.9, 19.2) | 16.38 (8.50, 29.25) | **< 0.001** |
| CRI-I | 3.37 (2.85, 3.98) | 3.66 (3.03, 4.28) | **< 0.001** |
| CRI-II | 2.03 (1.62, 2.55) | 2.28 (1.71, 2.71) | **< 0.001** |
| FBG (mmol/L) | 5.48 (4.89, 6.54) | 5.43 (4.82, 6.52) | 0.294 |
| TyG | 8.66 (8.26, 9.07) | 8.86 (8.47, 9.36) | **< 0.001** |
| UREA (mmol/L) | 5.97 (4.87, 7.15) | 6.26 (5.00, 8.20) | **< 0.001** |
| CREA (μmol/L) | 62.0 (52.1, 71.5) | 75.0 (63.6, 92.9) | **< 0.001** |
| UA_admission (μmol/L) | 278 (232, 321) | 453 (421, 495) | **< 0.001** |
| UA_3d (μmol/L) | 248 (195, 306) | 454 (398, 515) | **< 0.001** |
| K (mmol/L) | 3.87 (3.66, 4.09) | 3.89 (3.65, 4.17) | **0.036** |
| Na (mmol/L) | 140.9 (139.2, 142.2) | 140.8 (139.1, 142.0) | 0.366 |
| Cl (mmol/L) | 105.6 (104.0, 107.4) | 105.1 (103.3, 107.3) | **< 0.001** |
| PTA (%) | 111 (100, 124) | 112 (100, 126) | 0.375 |
| TT (s) | 16.3 (15.1, 17.4) | 16.7 (15.4, 17.7) | **< 0.001** |
| INR | 0.98 (0.94, 1.03) | 0.98 (0.93, 1.03) | 0.193 |
| APTT (s) | 27.9 (26.3, 30.1) | 28.2 (26.3, 30.4) | 0.122 |
| PT (s) | 11.0 (10.6, 11.5) | 11.0 (10.5, 11.4) | 0.543 |
| FIB (g/L) | 2.84 (2.41, 3.43) | 2.75 (2.32, 3.34) | **0.003** |

APT, antiplatelet therapy; SBP, systolic blood pressure; DBP, diastolic blood pressure; HTN, hypertension; AF, atrial fibrillation; CHD, coronary heart disease; HLP, hyperlipidemia; DM, diabetes mellitus; BMI, body mass index; NLR, neutrophil-to-lymphocyte ratio; LMR, lymphocyte-to-monocyte ratio; SII, systemic inflammatory index; PLR, platelet-to-lymphocyte ratio; HALP, hemoglobin, albumin, lymphocyte, platelet score; RBC, red blood cell; HGB, hemoglobin; HCT, hematocrit; MCHC, mean corpuscular hemoglobin concentration; RDW-CV, red blood cell distribution width-coefficient of variation; TC, total cholesterol; TG, triglyceride; LDL-C, low-density lipoprotein cholesterol; HDL-C, high-density lipoprotein cholesterol; non-HDL-C, non-high-density lipoprotein cholesterol; AIP, atherogenic index of plasma; AC, atherogenic coefficient; LCI, lipoprotein combine index; CRI-I, Castelli's index-I; CRI-II, Castelli's index-II; FBG, fasting blood glucose; TyG, triglyceride-glucose index; K, potassium; Na, sodium; UA, uric acid; PTA, prothrombin activity; TT, thrombin time; INR, international normalized ratio; APTT, activated partial thromboplastin time; PT, prothrombin time; FIB, fibrinogen.
