# Supplementary material for: Identifying metabolic parameters as key indicators of hyperuricemia and ischemic stroke comorbidity via interpretable Clinlabomics models
Source: Front Endocrinol (Lausanne). 2026 Jan 13;16:1737419. doi: 10.3389/fendo.2025.1737419 (PMC12834788; doi:10.3389/fendo.2025.1737419)
Supplement: Supplementary file 4 [file Table4.docx]

**Table S4 Basic characteristics of included participants stratified by comorbidity before PSM.**

| **Variables** | **Non-comorbidity**  **(N =3,541)** | **Comorbidity**  **(N = 1,082)** | ***P*** |
| --- | --- | --- | --- |
| Age (years) | 66 (56, 74) | 71 (60, 78) | < 0.001 |
| Gender (Male, n, %) | 1979 (56) | 676 (62) | < 0.001 |
| Marriage (Other status, n, %) | 348 (10) | 108 (10) | 0.928 |
| Nationality (Ethnic minority, n, %) | 169 (5) | 2 (0) | < 0.001 |
| APT, n (Yes, %) | 205 (6) | 225 (21) | < 0.001 |
| Antihypertensive therapy, n (Yes, %) | 1047 (30) | 723 (67) | < 0.001 |
| Antidiabetic therapy, n (Yes, %) | 338 (10) | 254 (23) | < 0.001 |
| Statins therapy, n (Yes, %) | 203 (6) | 232 (21) | < 0.001 |
| Urate-lowering therapy, n (Yes, %) | 25 (1) | 39 (4) | < 0.001 |
| Time of onset (h) | 48 (12, 144) | 48 (15, 168) | 0.088 |
| TOAST, n (%) |  |  | - |
| LAA | 358 (10) | 325 (30) |  |
| SAO | 580 (16) | 602 (56) |  |
| CE | 74 (2) | 89 (8) |  |
| SOE | 45 (1) | 47 (4) |  |
| SUE | 25 (1) | 19 (2) |  |
| GCS_admission | 1 (0,1) | 1 (0,1) | - |
| mRS_admission | 15 (15, 15) | 15 (15, 15) | - |
| NIHSS_admission | 2 (1, 4) | 1 (0, 3) | - |
| SBP (mmHg) | 135 (121, 149) | 139 (125, 157) | < 0.001 |
| DBP (mmHg) | 82 (73, 90) | 83 (74, 94) | < 0.001 |
| Drinking (Yes, n, %) | 500 (14) | 243 (22) | < 0.001 |
| Smoking (Yes, n, %) | 319 (9) | 124 (11) | 0.019 |
| HTN (Yes, n, %) | 1908 (54) | 795 (73) | < 0.001 |
| DM (Yes, n, %) | 582 (16) | 288 (27) | < 0.001 |
| AF (Yes, n, %) | 60 (2) | 57 (5) | < 0.001 |
| CHD (Yes, n, %) | 191 (5) | 86 (8) | 0.002 |
| HLP (Yes, n, %) | 1856 (52) | 678 (63) | < 0.001 |
| BMI (Kg/m^2) | 24.8 (22.77, 26.97) | 25.81 (24.03, 27.47) | < 0.001 |
| WBC (10^9/L) | 6.57 (5.4, 8) | 7.1 (5.8, 9.0) | < 0.001 |
| NEU (10^9/L) | 4.29 (3.24, 5.81) | 4.82 (3.71, 6.46) | < 0.001 |
| LYM (10^9/L) | 1.47 (1.09, 1.86) | 1.41 (1.03, 1.86) | 0.083 |
| MON (10^9/L) | 0.48 (0.36, 0.67) | 0.90 (0.47, 1.54) | < 0.001 |
| NLR | 2.88 (1.91, 4.56) | 3.27 (2.33, 5.19) | < 0.001 |
| LMR | 3.00 (1.64, 4.27) | 1.00 (1.00, 2.77) | < 0.001 |
| SII | 531 (344, 884) | 612 (395, 969) | < 0.001 |
| SIRI | 1.51 (0.82, 3.24) | 3.59 (1.70, 5.44) | < 0.001 |
| PNR | 43.28 (30.50, 60.04) | 38.69 (27.20, 50.80) | < 0.001 |
| PLR | 128.75 (96.3, 169.17) | 130.76 (95.38, 178.25) | 0.13 |
| MHR | 0.40 (0.28, 0.59) | 0.71 (0.39, 1.26) | < 0.001 |
| NHR | 3.39 (2.47, 4.79) | 3.96 (2.93, 5.53) | < 0.001 |
| PHR | 145.05 (110.16, 191.35) | 148.44 (112.31, 198.86) | 0.057 |
| HRR | 9.92 (8.64, 11.06) | 9.78 (8.42, 10.91) | 0.032 |
| HALP | 41.55 (28.72, 57.19) | 41.56 (27.87, 56.69) | 0.43 |
| RBC (10^12/L) | 4.32 (3.90, 4.76) | 4.36 (3.93, 4.79) | 0.062 |
| HGB (g/L) | 131 (118, 144) | 132 (118, 145) | 0.27 |
| HCT (%) | 39.7 (35.8, 43.4) | 39.85 (36.2, 43.8) | 0.291 |
| MCV (fL) | 92.5 (89.2, 95.5) | 92.1 (89.1, 95.2) | 0.092 |
| MCHC (g/L) | 331 (324, 337) | 331 (324, 338) | 0.654 |
| MCH (pg) | 30.6 (29.4, 31.8) | 30.5 (29.3, 31.7) | 0.168 |
| RDW-CV (%) | 13.3 (12.7, 14.0) | 13.6 (13.0, 14.3) | < 0.001 |
| PLT (10^9/L) | 186 (147, 225) | 187 (143, 232) | 0.483 |
| CRP (mg/L) | 3.70 (1.03, 6.28) | 3.43 (1.01, 14.24) | < 0.001 |
| TC (mmol/L) | 4.46 (3.73, 5.20) | 4.46 (3.64, 5.34) | 0.863 |
| TG (mmol/L) | 1.40 (0.97, 2.03) | 1.64 (1.11, 2.38) | < 0.001 |
| LDL-C (mmol/L) | 2.67 (2.16, 3.25) | 2.73 (2.09, 3.35) | 0.305 |
| HDL-C (mmol/L) | 1.25 (1.06, 1.49) | 1.22 (1.04, 1.45) | 0.004 |
| Non-HDL-C (mmol/L) | 3.15 (2.51, 3.86) | 3.20 (2.45, 4.01) | 0.451 |
| AIP | 0.04 (-0.13, 0.24) | 0.12 (-0.06, 0.34) | < 0.001 |
| AC | 2.56 (1.94, 3.20) | 2.61 (1.99, 3.33) | 0.014 |
| LCI | 13.55 (7.27, 23.63) | 16.16 (8.00, 31.89) | < 0.001 |
| CRI-I | 3.56 (2.94, 4.20) | 3.61 (2.99, 4.33) | 0.014 |
| CRI-II | 2.14 (1.68, 2.68) | 2.22 (1.70, 2.77) | 0.005 |
| FBG (mmol/L) | 5.67 (4.94, 7.20) | 6.40 (5.31, 8.43) | < 0.001 |
| TyG | 8.81 (8.38, 9.27) | 9.08 (8.61, 9.62) | < 0.001 |
| UREA (mmol/L) | 6.03 (4.85, 7.50) | 6.86 (5.44, 9.03) | < 0.001 |
| CREA (μmol/L) | 68.3 (57.4, 82.4) | 85.9 (69.7, 110.6) | < 0.001 |
| UA_admission (μmol/L) | 342 (265, 430) | 458 (425, 514) | < 0.001 |
| UA_3d (μmol/L) | 332 (237, 430) | 464 (413, 513) | < 0.001 |
| K (mmol/L) | 3.86 (3.63, 4.11) | 3.87 (3.58, 4.17) | 0.561 |
| Na (mmol/L) | 140.6 (139.0, 142.1) | 140.4 (138.6, 142.1) | 0.053 |
| Cl (mmol/L) | 105.2 (103.2, 107.1) | 104.4 (102.0, 106.6) | < 0.001 |
| PTA (%) | 112 (99, 125) | 113 (98, 127) | 0.835 |
| TT (s) | 16.7 (15.5, 17.7) | 17.1 (16.0, 18.2) | < 0.001 |
| INR | 0.98 (0.93, 1.03) | 0.97 (0.92, 1.03) | 0.061 |
| APTT (s) | 28.1 (26.3, 30.4) | 28.3 (26.6, 30.3) | 0.365 |
| PT (s) | 11.0 (10.5, 11.6) | 11.1 (10.6, 11.8) | < 0.001 |
| FIB (g/L) | 2.88 (2.43, 3.52) | 3.12 (2.55, 3.83) | < 0.001 |

PSM, propensity score matching; APT, antiplatelet therapy; SBP, systolic blood pressure; DBP, diastolic blood pressure; TOAST, Trial of Org 10172 in Acute Stroke Treatment; LAA, large-artery atherosclerosis; SAO, small artery occlusion; CE, Cardioembolism; SOE, stroke of other determined etiologies; SUE, stroke of undetermined etiologies; GCS, Glasgow Coma Scale; mRS, Modified Rankin Scale; NIHSS, National Institutes of Health Stroke Scale; HTN, hypertension; AF, atrial fibrillation; CHD, coronary heart disease; HLP, hyperlipidemia; DM, diabetes mellitus; BMI, body mass index; NLR, neutrophil-to-lymphocyte ratio; LMR, lymphocyte-to-monocyte ratio; SII, systemic inflammatory index; PLR, platelet-to-lymphocyte ratio; HALP, hemoglobin, albumin, lymphocyte, platelet score; RBC, red blood cell; HGB, hemoglobin; HCT, hematocrit; MCHC, mean corpuscular hemoglobin concentration; RDW-CV, red blood cell distribution width-coefficient of variation; TC, total cholesterol; TG, triglyceride; LDL-C, low-density lipoprotein cholesterol; HDL-C, high-density lipoprotein cholesterol; non-HDL-C, non-high-density lipoprotein cholesterol; AIP, atherogenic index of plasma; AC, atherogenic coefficient; LCI, lipoprotein combine index; CRI-I, Castelli's index-I; CRI-II, Castelli's index-II; FBG, fasting blood glucose; TyG, triglyceride-glucose index; K, potassium; Na, sodium; UA, uric acid; PTA, prothrombin activity; TT, thrombin time; INR, international normalized ratio; APTT, activated partial thromboplastin time; PT, prothrombin time; FIB, fibrinogen.
