# Supplementary material for: Identifying metabolic parameters as key indicators of hyperuricemia and ischemic stroke comorbidity via interpretable Clinlabomics models
Source: Front Endocrinol (Lausanne). 2026 Jan 13;16:1737419. doi: 10.3389/fendo.2025.1737419 (PMC12834788; doi:10.3389/fendo.2025.1737419)
Supplement: Supplementary file 5 [file Table5.docx]

**Table S5 Differential indicators among three groups and their intersection**

| Comorbidity vs. Non-comorbidity (n=49) | HUA HCs vs. Non-HUA HCs (n=49) | Non-HUA IS vs. Non-HUA HCs (n=57) | Intersection  (n=33) |
| --- | --- | --- | --- |
| Age | Age | Age | Age |
| Gender | Gender | Gender | Gender |
| Nationality | Marriage | Marriage | Nationality |
| APT | Nationality | Nationality | Antihypertensive therapy |
| Antihypertensive therapy | Antihypertensive therapy | APT | Antidiabetic therapy |
| Antidiabetic therapy | Antidiabetic therapy | Antihypertensive therapy | Smoking |
| Statins therapy | Urate-lowering therapy | Antidiabetic therapy | Drinking |
| Urate-lowering therapy | DBP | Statins therapy | DBP |
| NIHSS_admission | Drinking | SBP | HTN |
| SBP | Smoking | DBP | AF |
| DBP | HTN | Drinking | CHD |
| Drinking | AF | Smoking | HLP |
| Smoking | CHD | HTN | BMI |
| HTN | HLP | DM | WBC |
| DM | BMI | AF | NEU |
| AF | WBC | CHD | MON |
| CHD | NEU | HLP | PNR |
| HLP | LYM | BMI | NHR |
| BMI | MON | WBC | MHR |
| WBC | PNR | NEU | TyG |
| NEU | PLR | LYM | TG |
| MON | MHR | MON | HDL-C |
| NLR | NHR | NLR | AIP |
| LMR | PHR | LMR | LCI |
| SII | HRR | SII | AC |
| SIRI | HALP | SIRI | CRI-I |
| PNR | RBC | PNR | CRI-II |
| MHR | HGB | PLR | Cl |
| NHR | HCT | MHR | CREA |
| HRR | MCV | NHR | UA_admission |
| RDW-CV | MCHC | PHR | UA_3d |
| CRP | MCH | HALP | TT |
| TG | TG | MCV | FIB |
| HDL-C | HDL-C | MCHC |  |
| AIP | Non-HDL-C | RDW-CV |  |
| AC | AIP | PLT |  |
| LCI | AC | CRP |  |
| CRI-I | LCI | TG |  |
| CRI-II | CRI-I | HDL-C |  |
| FBG | CRI-II | AIP |  |
| TyG | TyG | AC |  |
| UREA | UREA | LCI |  |
| CREA | CREA | CRI-I |  |
| UA_admission | UA_admission | CRI-II |  |
| UA_3d | UA_3d | FBG |  |
| Cl | K | TyG |  |
| TT | Cl | CREA |  |
| PT | TT | UA_admission |  |
| FIB | FIB | UA_3d |  |
|  |  | K |  |
|  |  | Na |  |
|  |  | Cl |  |
|  |  | TT |  |
|  |  | INR |  |
|  |  | APTT |  |
|  |  | PT |  |
|  |  | FIB |  |
