# Supplementary material for: Identifying metabolic parameters as key indicators of hyperuricemia and ischemic stroke comorbidity via interpretable Clinlabomics models
Source: Front Endocrinol (Lausanne). 2026 Jan 13;16:1737419. doi: 10.3389/fendo.2025.1737419 (PMC12834788; doi:10.3389/fendo.2025.1737419)
Supplement: Supplementary file 6 [file Table6.docx]

**Table S6 The SMD values between pre-PSM and post-PSM groups.**

| Covariate | Pre_PSM_SMD | Post_PSM_SMD |
| --- | --- | --- |
| distance | 0.883660881 | 0.025585605 |
| gender | 0.065887275 | 0.013579049 |
| age | 0.42335504 | -0.032531872 |
| drinking | 0.083381054 | 0.006789525 |
| smoking | 0.024515042 | 0.009699321 |
| HTN | 0.195919623 | -0.003879728 |
| DM | 0.101813402 | 0.002909796 |
| AF | 0.035735856 | 0.013579049 |
| CHD | 0.025542875 | 0.003879728 |
| HLP | 0.102471654 | 0.032977692 |
| BMI | 0.247652134 | -0.029527796 |
| APT | 0.150054993 | 0.022308438 |
| Antihypertensive therapy | 0.372527837 | -0.013579049 |
| Antidiabetic therapy | 0.1392972 | 0.004849661 |
| Statins therapy | 0.157089307 | 0.016488846 |
| Urate lowering therapy | 0.028701804 | 0.001939864 |
| WBC | 0.220641605 | 0.020109379 |
| SII | 0.17693214 | 0.010148219 |

SMD, standardized mean difference; PSM, propensity score matching.
