# Supplementary material for: Identifying metabolic parameters as key indicators of hyperuricemia and ischemic stroke comorbidity via interpretable Clinlabomics models
Source: Front Endocrinol (Lausanne). 2026 Jan 13;16:1737419. doi: 10.3389/fendo.2025.1737419 (PMC12834788; doi:10.3389/fendo.2025.1737419)
Supplement: Supplementary file 7 [file Table7.docx]

**Table S7 The associations between metabolic parameters and the risk of HUA-IS comorbidity before and following PSM.**

| Variables | Before PSM | | | | Following PSM | | | |
| --- | --- | --- | --- | --- | --- | --- | --- | --- |
|  | Univariate | *P*^1^ | Multivariate | *P*^2^ | Univariate | *P*^1^ | Multivariate | *P*^2^ |
| UA_admission | 1.01 (1.01-1.01) | **< 0.001** | 1.02 (1.02-1.02) | **< 0.001** | 1.02 (1.02-1.03) | **< 0.001** | 1.03 (1.00-1.06) | 0.069 |
| UA_3d | 1.01 (1.01-1.01) | **< 0.001** | 1.01 (1.01-1.01) | **< 0.001** | 1.02 (1.02-1.02) | **< 0.001** | 1.02 (1.02-1.02) | **< 0.001** |
| TyG | 1.60 (1.47-1.76) | **< 0.001** | 1.32 (1.17-1.48) | **< 0.001** | 1.31 (1.16-1.47) | **< 0.001** | 1.40 (1.21-1.62) | **< 0.001** |
| TG | 1.07 (1.03-1.11) | **< 0.001** | 1.05 (1.01-1.09) | **0.027** | 1.13 (1.05-1.20) | **< 0.001** | 1.13 (1.05-1.22) | **0.002** |
| HDL-C | 0.74 (0.60-0.90) | **0.003** | 0.96 (0.76-1.22) | 0.748 | 1.00 (0.78-1.30) | 0.977 | 1.08 (0.81-1.44) | 0.610 |
| AIP | 2.37 (1.88-2.98) | **< 0.001** | 2.21 (1.59-3.07) | **< 0.001** | 2.02 (1.49-2.75) | **< 0.001** | 2.74 (1.80-4.19) | **< 0.001** |
| AC | 1.08 (1.02-1.15) | **0.014** | 1.05 (0.98-1.14) | 0.189 | 1.10 (1.01-1.20) | **0.034** | 1.10 (0.99-1.22) | 0.079 |
| LCI | 1.00 (1.00-1.00) | **0.006** | 1.00 (1.00-1.00) | **0.022** | 1.01 (1.00-1.01) | **< 0.001** | 1.01 (1.00-1.01) | **0.001** |
| CRI-I | 1.08 (1.02-1.15) | **0.014** | 1.05 (0.98-1.14) | 0.189 | 1.10 (1.01-1.20) | **0.034** | 1.10 (0.99-1.22) | 0.079 |
| CRI-II | 1.16 (1.06-1.27) | **0.001** | 1.11 (1.00-1.24) | 0.055 | 1.14 (1.01-1.28) | **0.033** | 1.13 (0.99-1.30) | 0.073 |

PSM, propensity score matching; UA, uric acid; TyG, triglyceride-glucose index; TG, triglyceride; HDL-C, high-density lipoprotein cholesterol; AIP, atherogenic index of plasma; AC, atherogenic coefficient; LCI, lipoprotein combine index; CRI-I, Castelli's index-I; CRI-II, Castelli's index-II; *P*^1^, the *P*-value from univariate analysis; *P*^2^, the *P*-value from multivariate analysis.
