# Supplementary material for: Identifying metabolic parameters as key indicators of hyperuricemia and ischemic stroke comorbidity via interpretable Clinlabomics models
Source: Front Endocrinol (Lausanne). 2026 Jan 13;16:1737419. doi: 10.3389/fendo.2025.1737419 (PMC12834788; doi:10.3389/fendo.2025.1737419)
Supplement: Supplementary file 8 [file Table8.docx]

**Table S8 Quartile stratification of metabolic indicators significantly associated with comorbidities.**

| Variables | Q1 | Q2 | Q3 | Q4 |
| --- | --- | --- | --- | --- |
| UA_3d (μmol/L) | ≤ 264 | 264 - 366 | 366 - 466 | > 466 |
| TyG | ≤ 8.43 | 8.43 - 8.86 | 8.86 - 9.36 | > 9.36 |
| TG (mmol/L) | ≤ 1 | 1 - 1.45 | 1.45 - 2.13 | > 2.13 |
| AIP | ≤ -0.113 | -0.113 - 0.065 | 0.065 - 0.267 | > 0.267 |
| LCI | ≤ 7.41 | 7.41 - 14 | 14 - 25.9 | > 25.9 |
