# Supplementary material for: Identifying metabolic parameters as key indicators of hyperuricemia and ischemic stroke comorbidity via interpretable Clinlabomics models
Source: Front Endocrinol (Lausanne). 2026 Jan 13;16:1737419. doi: 10.3389/fendo.2025.1737419 (PMC12834788; doi:10.3389/fendo.2025.1737419)
Supplement: Supplementary file 9 [file Table9.docx]

**Table S9 The associations between metabolic parameters and the risk of HUA-IS comorbidity.**

| Variables |  | PSM | Q1 | Q2 | Q3 | Q4 | Pa | Pb | Pc | P for trend |
| --- | --- | --- | --- | --- | --- | --- | --- | --- | --- | --- |
| UA_3d | M1 | Before | Ref | 9.0×10^6(0-1.5×10^148) | 8.1×10^7 (0-1.4×10^149) | 9.5×10^7 (0-1.6×10^149) | 0.923 | 0.913 | 0.912 | - |
|  | M2 | Before | Ref | 2.5×10^7 (0-3.4×10^221) | 2.8×10^8 (0-3.9×10^222) | 3.5×10^8 (0-4.9×10^222) | 0.946 | 0.938 | 0.938 | - |
|  | M3 | Before | Ref | 3.4×10^7 (0-2.2×10^205) | 5.9×10^ 8(0-3.8×10^206) | 7.9×10^8 (0-5.2×10^206) | 0.941 | 0.931 | 0.930 | - |
| TyG | M1 | Before | Ref | 1.51 (1.22-1.87) | 1.79 (1.45-2.21) | 2.82 (2.30-3.45) | < 0.001 | < 0.001 | < 0.001 | < 0.001 |
|  | M2 | Before | Ref | 1.36 (1.09-1.69) | 1.50 (1.19-1.89) | 2.16 (1.68-2.77) | 0.006 | 0.001 | < 0.001 | < 0.001 |
|  | M3 | Before | Ref | 1.29 (1.03-1.62) | 1.31 (1.03-1.67) | 1.80 (1.39-2.33) | 0.027 | 0.025 | < 0.001 | < 0.001 |
| TG | M1 | Before | Ref | 1.53 (1.24-1.88) | 1.58 (1.29-1.94) | 2.22 (1.82-2.71) | < 0.001 | < 0.001 | < 0.001 | < 0.001 |
|  | M2 | Before | Ref | 1.50 (1.21-1.86) | 1.55 (1.23-1.95) | 2.07 (1.61-2.67) | < 0.001 | < 0.001 | < 0.001 | < 0.001 |
|  | M3 | Before | Ref | 1.47 (1.18-1.83) | 1.49 (1.18-1.90) | 2.07 (1.59-2.70) | 0.001 | 0.001 | < 0.001 | < 0.001 |
| AIP | M1 | Before | Ref | 1.14 (0.93-1.41) | 1.44 (1.18-1.76) | 1.96 (1.61-2.38) | 0.201 | < 0.001 | < 0.001 | < 0.001 |
|  | M2 | Before | Ref | 1.05 (0.84-1.31) | 1.29 (1.00-1.65) | 1.84 (1.40-2.42) | 0.656 | 0.047 | < 0.001 | < 0.001 |
|  | M3 | Before | Ref | 1.05 (0.84-1.31) | 1.26 (0.98-1.63) | 1.76(1.33-2.34) | 0.684 | 0.076 | < 0.001 | < 0.001 |
| LCI | M1 | Before | Ref | 0.91 (0.74-1.11) | 1.06 (0.87-1.30) | 1.61(1.33-1.94) | 0.339 | 0.536 | < 0.001 | < 0.001 |
|  | M2 | Before | Ref | 0.98 (0.80-1.21) | 1.08 (0.87-1.34) | 1.56 (1.23-1.99) | 0.869 | 0.508 | < 0.001 | < 0.001 |
|  | M3 | Before | Ref | 0.98 (0.79-1.22) | 1.09 (0.87-1.37) | 1.56 (1.21-2.00) | 0.858 | 0.439 | 0.001 | 0.001 |
| UA_3d | M1 | After | Ref | 2.6×10^7 (0-4.9×10^248) | 3.7×10^8 (0-6.9×10^249) | 5.0×10^8 (0-9.4×10^249) | 0.952 | 0.945 | 0.944 | - |
|  | M2 | After | Ref | 2.9×10^7 (0-1.1×10^243) | 5.4×10^8 (0-2.1×10^244) | 7.6×10^8 (0-2.9×10^244) | 0.951 | 0.942 | 0.941 | - |
|  | M3 | After | Ref | 3.2×10^7 (0-3.1×10^238) | 6.8×10^8 (0-6.4×10^239) | 9.5×10^8 (0-9.0×10^239) | 0.949 | 0.940 | 0.939 | - |
| TyG | M1 | After | Ref | 1.23 (0.94-1.61) | 1.32 (1.01-1.72) | 1.72 (1.33-2.22) | 0.130 | 0.040 | < 0.001 | < 0.001 |
|  | M2 | After | Ref | 1.22 (0.93-1.61) | 1.39 (1.04-1.85) | 1.90 (1.40-2.59) | 0.152 | 0.025 | < 0.001 | < 0.001 |
|  | M3 | After | Ref | 1.23 (0.93-1.62) | 1.40 (1.05-1.87) | 1.92 (1.40-2.62) | 0.145 | 0.023 | < 0.001 | < 0.001 |
| TG | M1 | After | Ref | 1.47 (1.14-1.90) | 1.50 (1.16-1.93) | 2.09 (1.63-2.69) | 0.003 | 0.002 | < 0.001 | < 0.001 |
|  | M2 | After | Ref | 1.54 (1.19-2.00) | 1.74 (1.31-2.31) | 2.59 (1.90-3.54) | 0.001 | < 0.001 | < 0.001 | < 0.001 |
|  | M3 | After | Ref | 1.55 (1.19-2.02) | 1.74 (1.31-2.32) | 2.60 (1.90-3.56) | 0.001 | < 0.001 | < 0.001 | < 0.001 |
| AIP | M1 | After | Ref | 1.02 (0.79-1.32) | 1.20 (0.94-1.55) | 1.58 (1.23-2.02) | 0.887 | 0.150 | < 0.001 | < 0.001 |
|  | M2 | After | Ref | 1.13 (0.87-1.47) | 1.45 (1.06-1.97) | 2.00 (1.43-2.81) | 0.364 | 0.019 | < 0.001 | < 0.001 |
|  | M3 | After | Ref | 1.14 (0.87-1.49) | 1.45 (1.06-1.97) | 2.00 (1.42-2.81) | 0.340 | 0.020 | < 0.001 | < 0.001 |
| LCI | M1 | After | Ref | 0.86 (0.67-1.11) | 1.04 (0.81-1.33) | 1.50 (1.17-1.91) | 0.244 | 0.757 | 0.001 | < 0.001 |
|  | M2 | After | Ref | 0.89 (0.69-1.15) | 1.12 (0.86-1.47) | 1.67 (1.24-2.25) | 0.377 | 0.403 | 0.001 | < 0.001 |
|  | M3 | After | Ref | 0.89 (0.69-1.15) | 1.13 (0.86-1.48) | 1.68 (1.25-2.27) | 0.372 | 0.388 | 0.001 | < 0.001 |

TyG, triglyceride-glucose index; TG, triglyceride; AIP, atherogenic index of plasma; LCI, lipoprotein combine index; P^a^, the P-value from Q2 vs. Q1; P^b^, the P-value from Q3 vs. Q1; P^c^, the P-value from Q4 vs. Q1.
