# Supplementary figures and images for: Identifying metabolic parameters as key indicators of hyperuricemia and ischemic stroke comorbidity via interpretable Clinlabomics models
Source: Front Endocrinol (Lausanne). 2026 Jan 13;16:1737419. doi: 10.3389/fendo.2025.1737419 (PMC12834788; doi:10.3389/fendo.2025.1737419)

- Comorbidity vs. Non-comorbidity
- HUA HCs vs. Non-HUA HCs
- Non-HUA IS vs. Non-HUA HCs

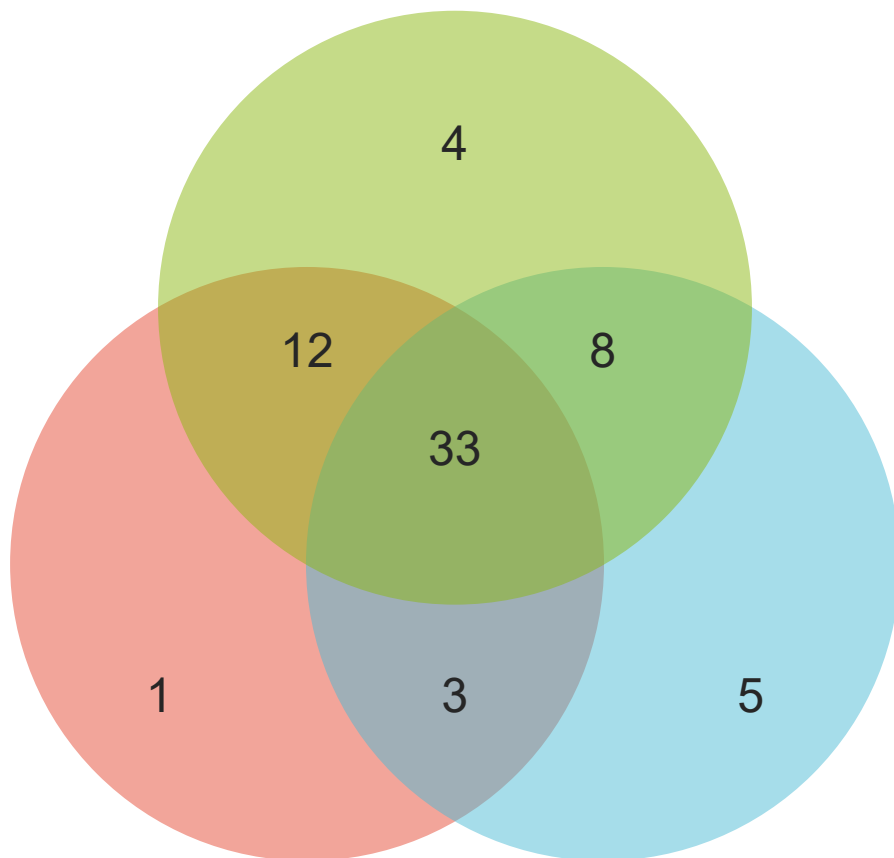

Supplement: Supplementary file 10 [file Image1.pdf]

# Covariate Balance

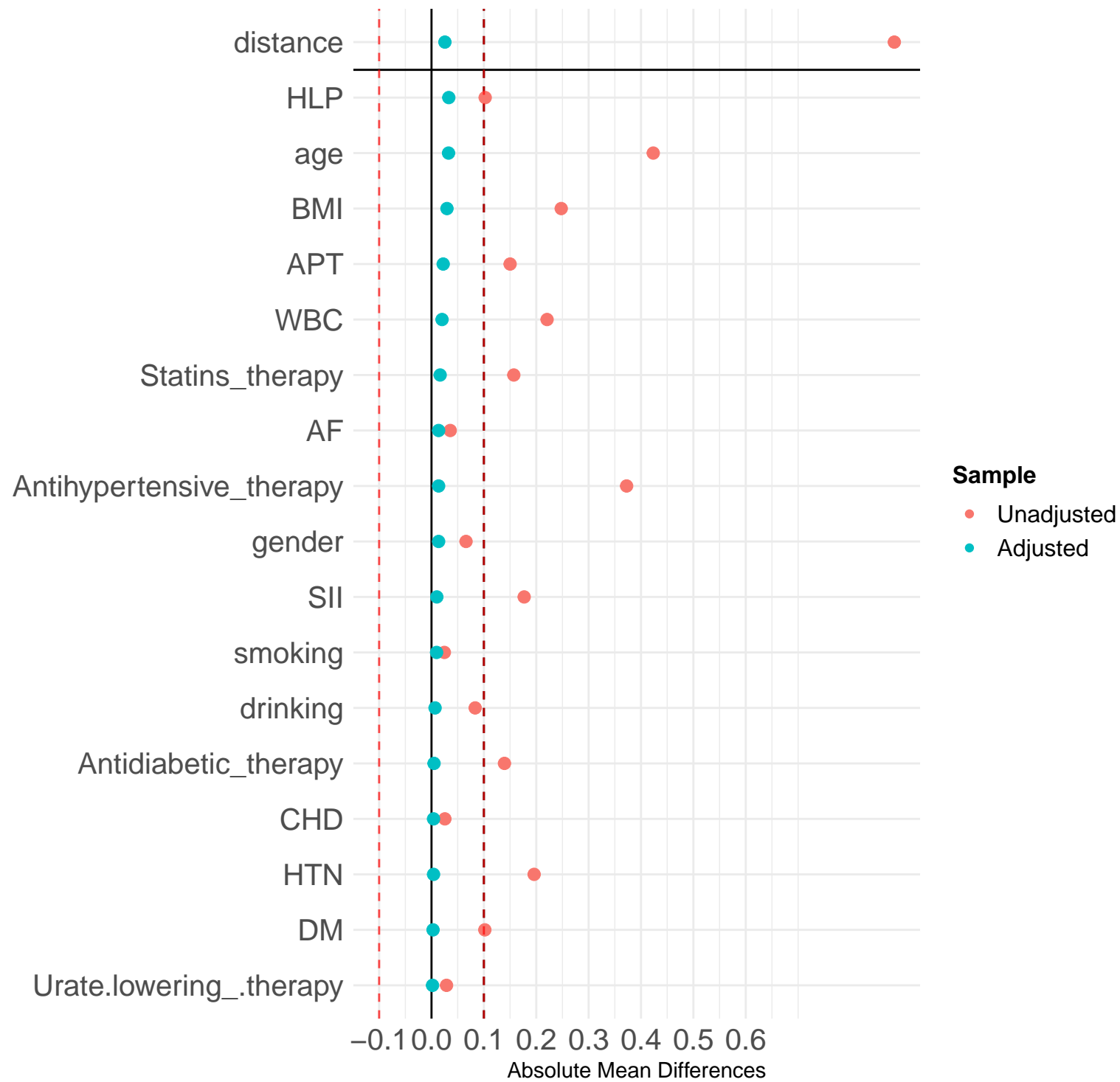

Supplement: Supplementary file 11 [file Image2.pdf]

**A**

Predicted Probability

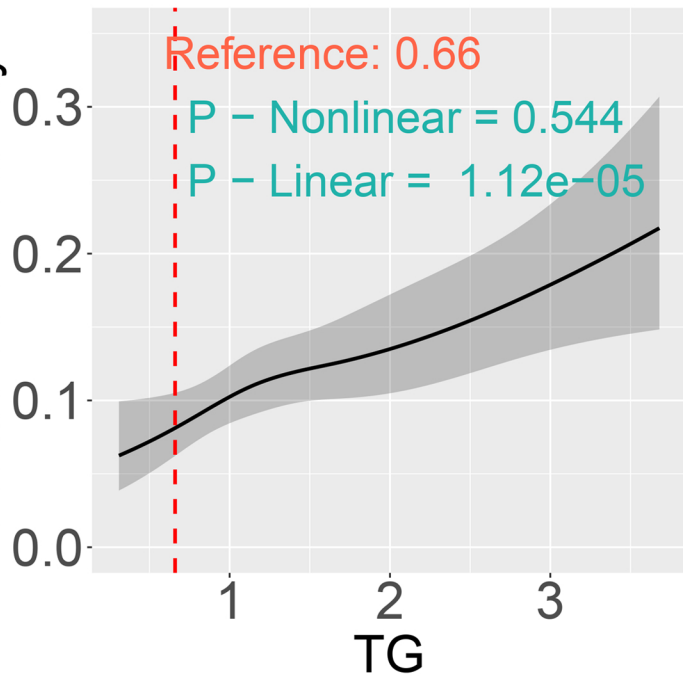**B**

Predicted Probability

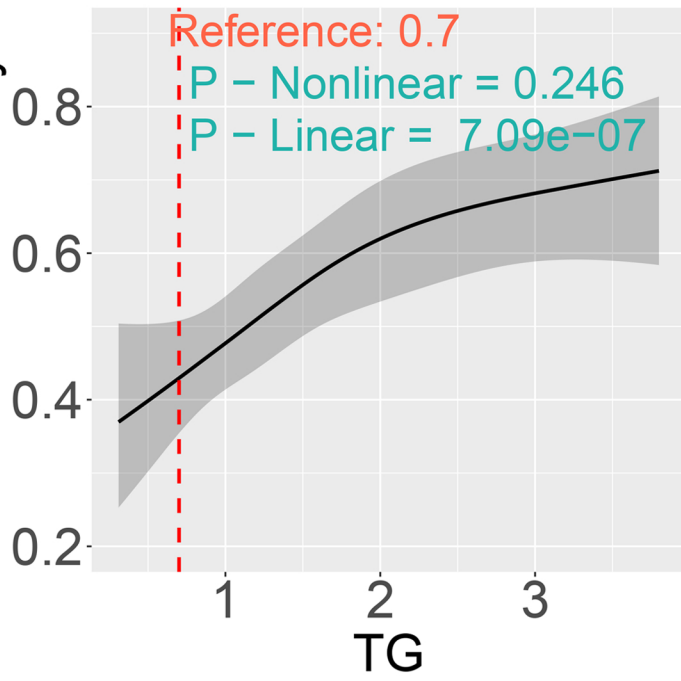

Supplement: Supplementary file 12 [file Image3.pdf]

**A**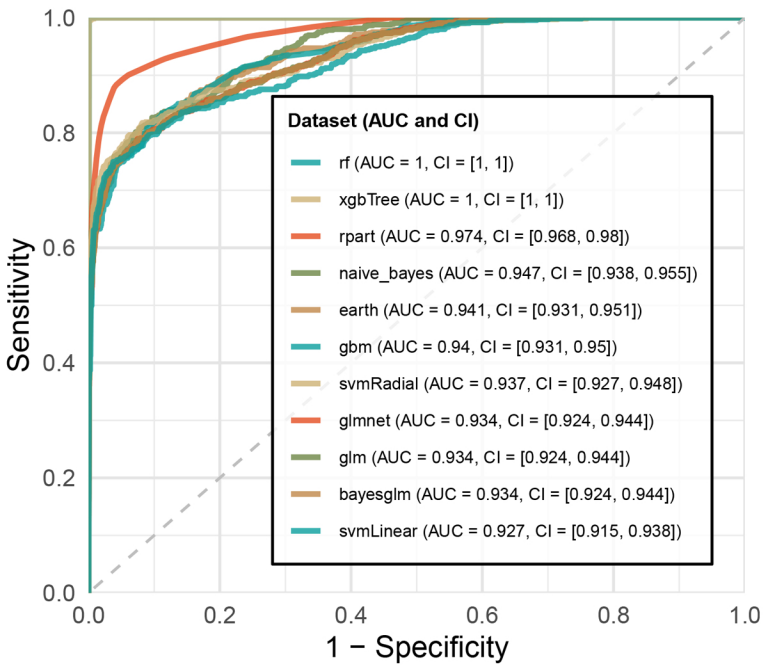**B**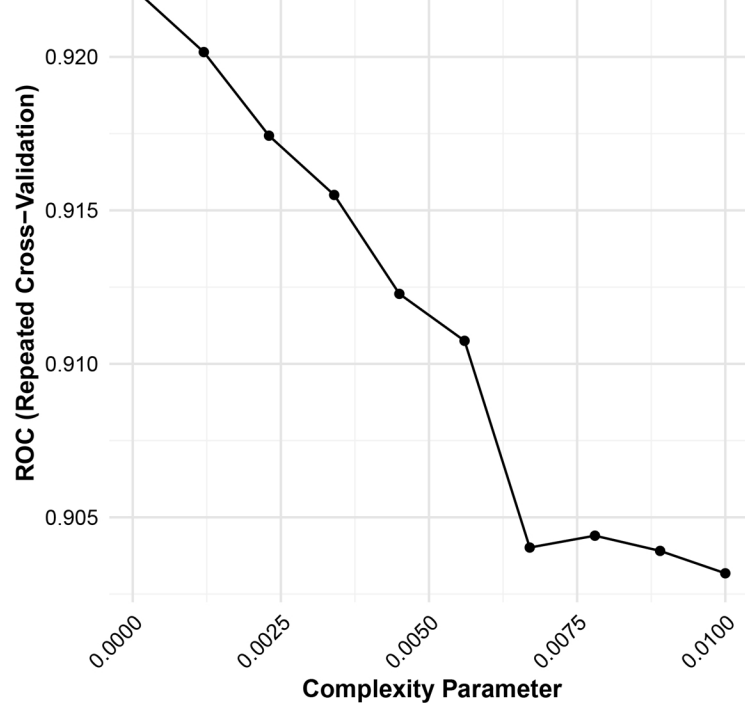**C**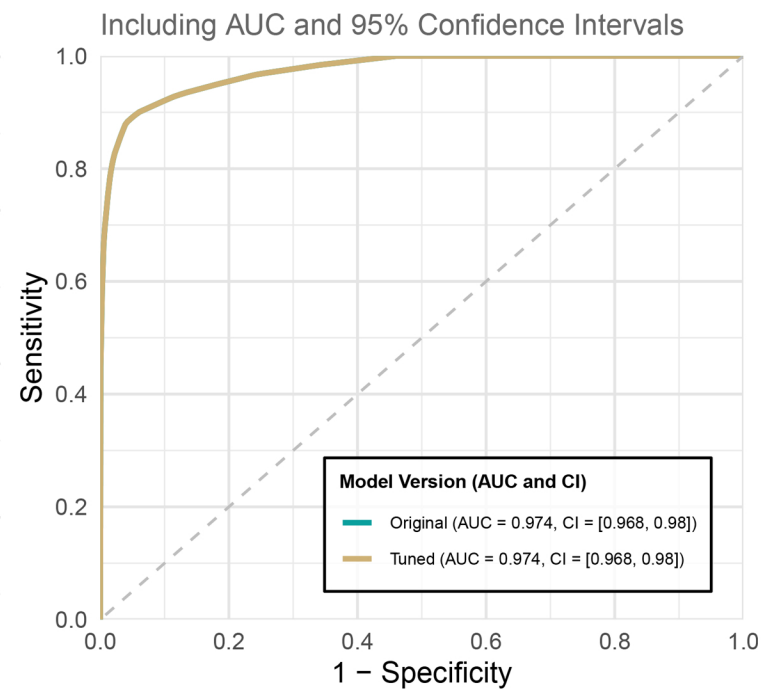

Supplement: Supplementary file 13 [file Image4.pdf]

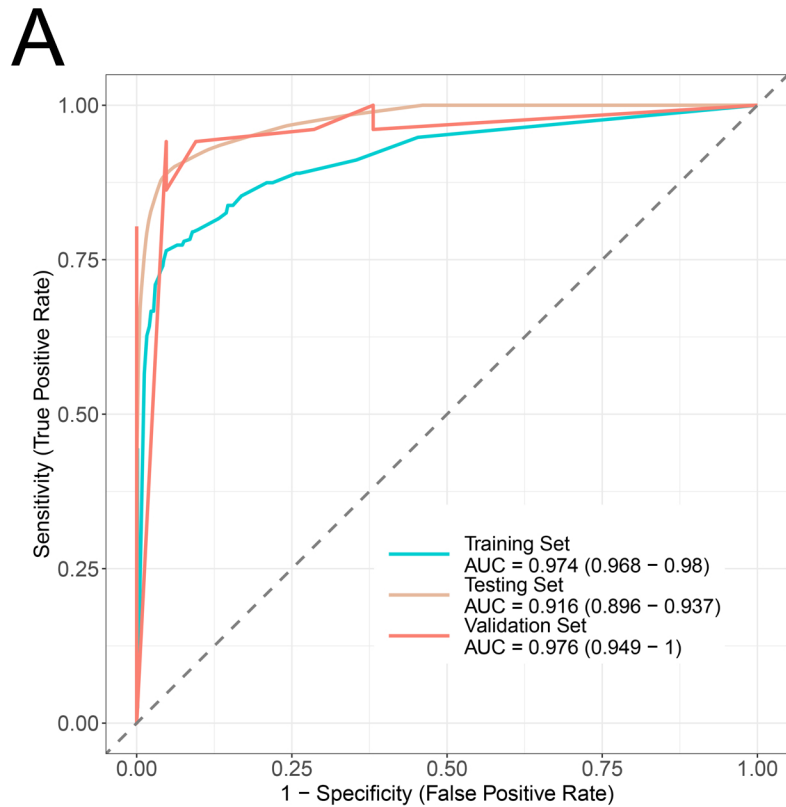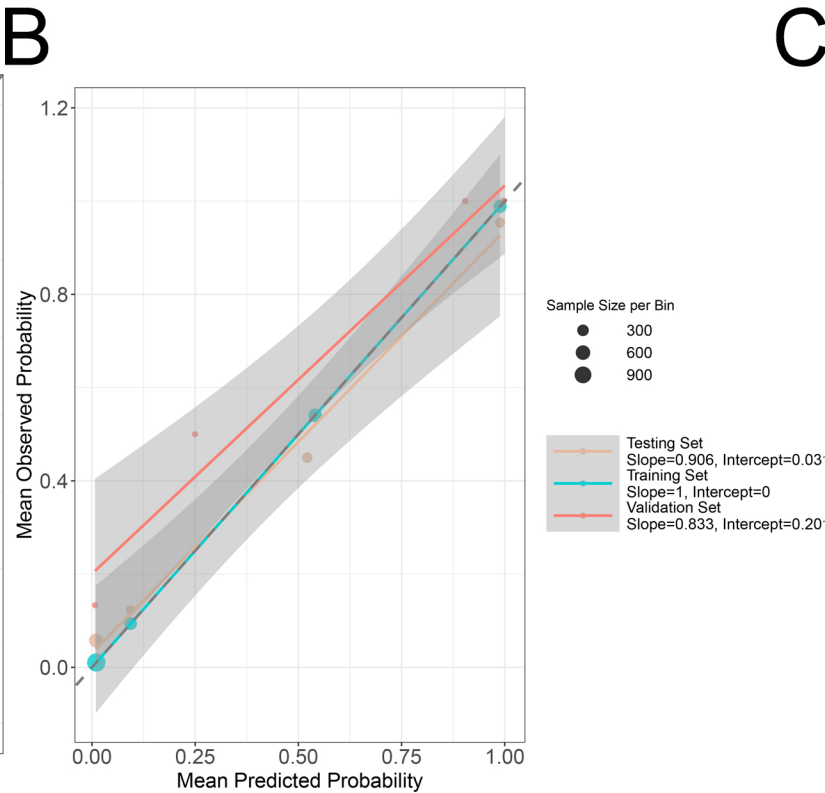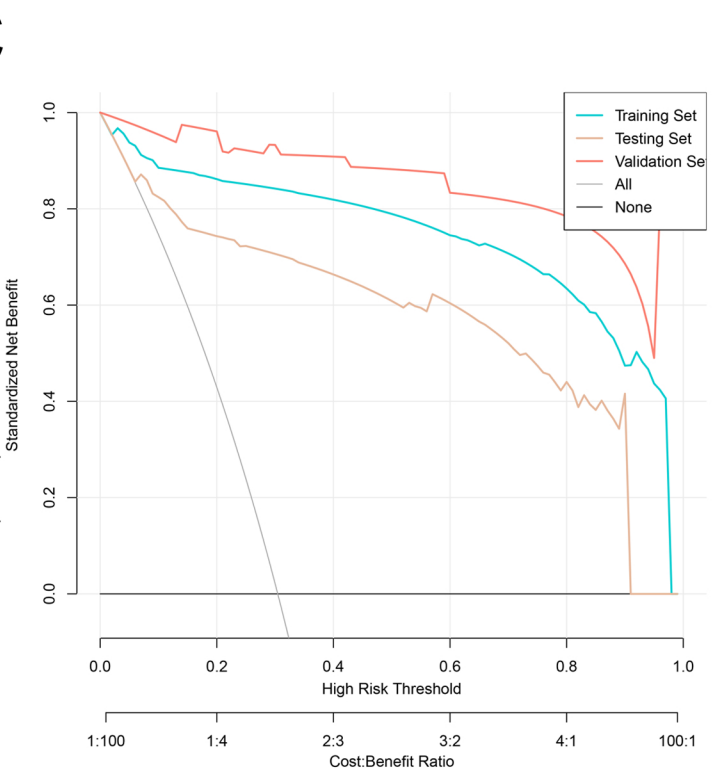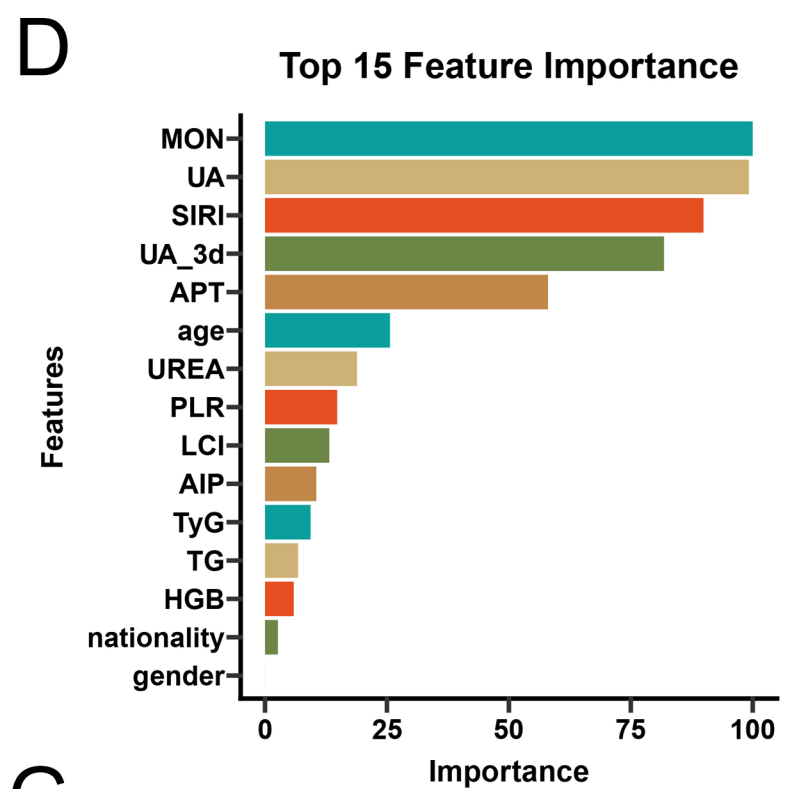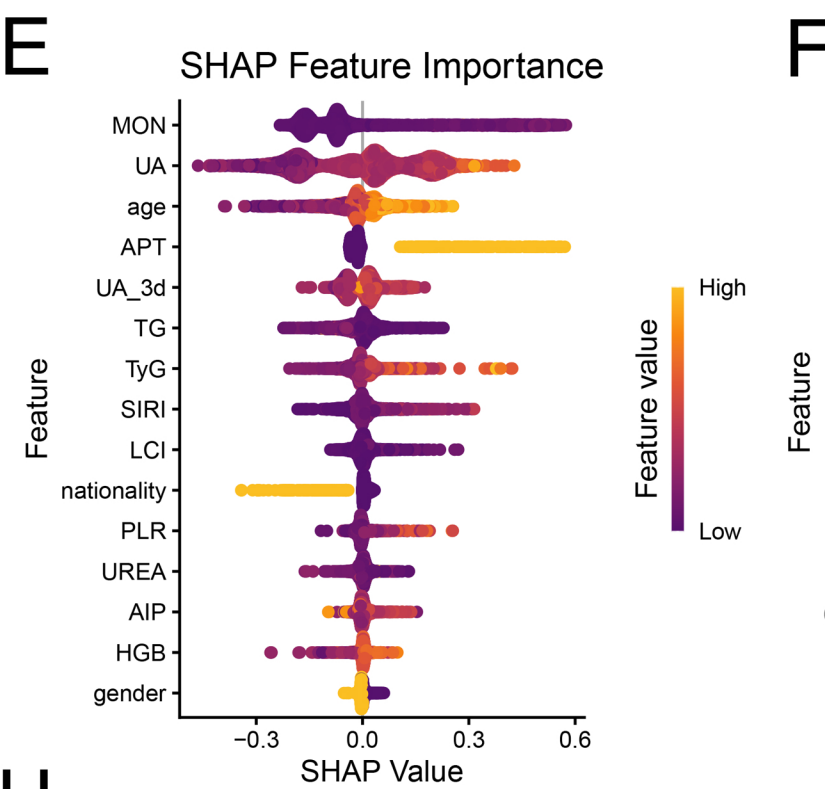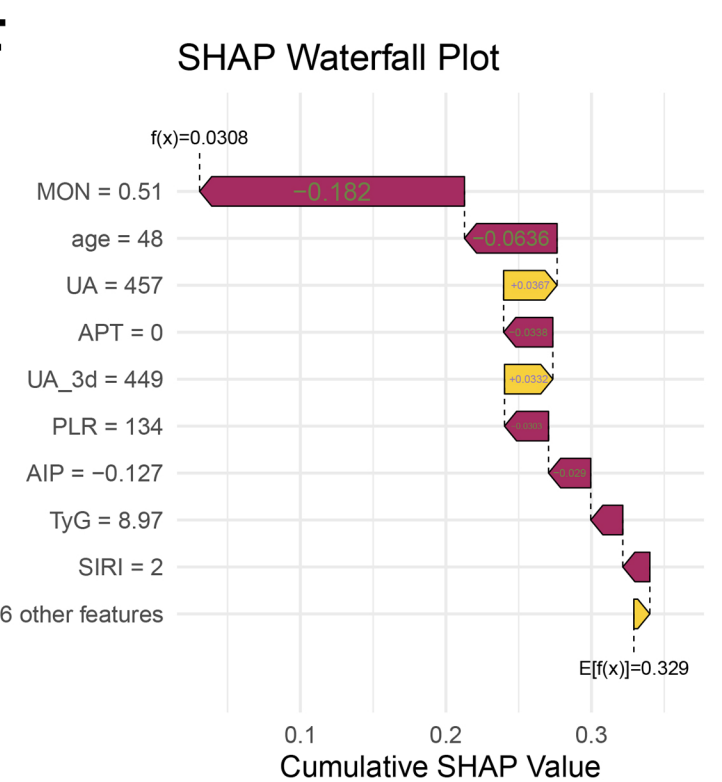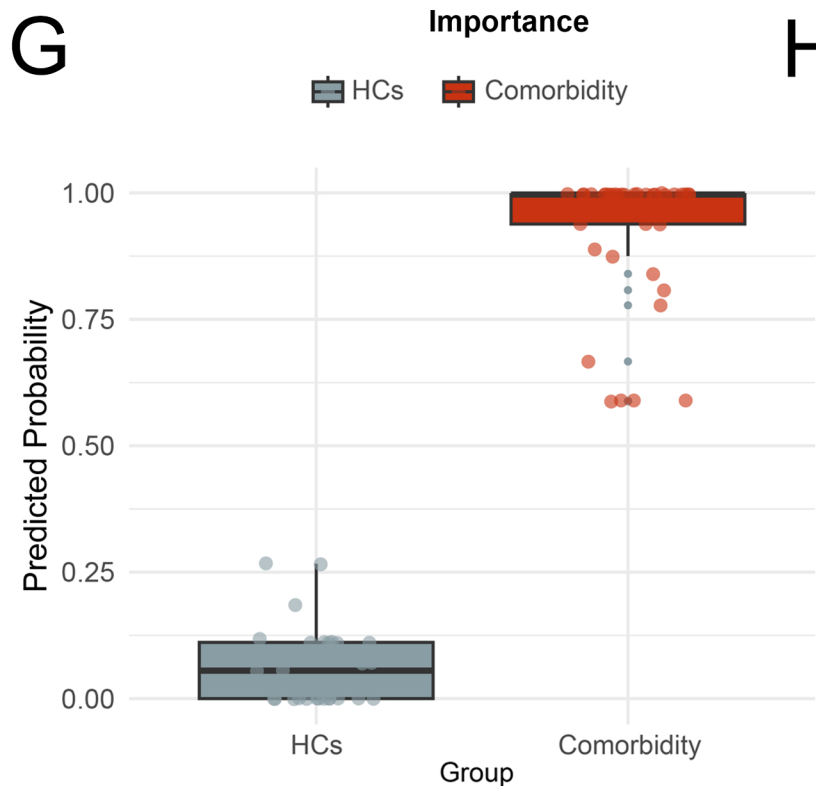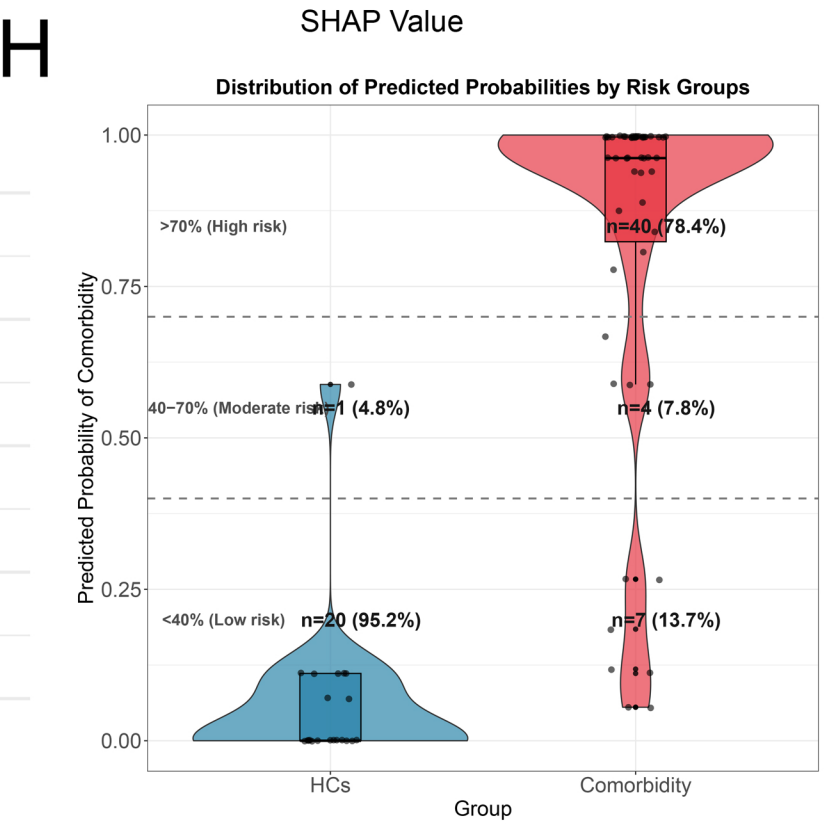

Supplement: Supplementary file 14 [file Image5.pdf]
